# Supplementary material for: Controllable protein design via autoregressive direct coupling analysis conditioned on principal components
Source: PLoS Comput Biol. 2026 Feb 19;22(2):e1013996. doi: 10.1371/journal.pcbi.1013996 (PMC12935303; doi:10.1371/journal.pcbi.1013996)
Supplement: S1 File — Including: Appendix A1: Direct Coupling Analysis Details; Appendix A2: Mathematical foundation of the feature-conditioned autoregressive model; Appendix A3: Implementation details; Appendix A4: data processing and MSA construction; Appendix A5: Wasserstein distance and Sinkhorn divergence; Appendix A6: Principal components higher than the second; Appendix A7: Additional structural analysis of the PF00014 mismatch case; Appendix A8: pLDDT analysis for generated RR homodimer sequences; Appendix A9: In-silico deep mutational scanning; Appendix A10: Supplementary figures. (PDF) [file pcbi.1013996.s001.pdf]

Supplementary Material: Controllable Protein Design via Autoregressive Direct Coupling Analysis Conditioned on Principal Components

Francesco Caredda,[1,*] Lisa Gennai,[2] Paolo De Los Rios,[3,4] and Andrea Pagnani[1,5,6]

[1] *Department of Applied Science and Technology, Politecnico di Torino, Torino, Italy.*
[2] *Institute of Physics, School of Basic Sciences, École Polytechnique Fédérale de Lausanne - EPFL, Lausanne, CH-1015, Switzerland*
[3] *Institute of Physics, School of Basic Sciences, École Polytechnique Fédérale de Lausanne - EPFL, Lausanne, Switzerland*
[4] *Institute of Bioengineering, School of Basic Sciences, École Polytechnique Fédérale de Lausanne - EPFL, Lausanne, Switzerland*
[5] *Italian Institute for Genomic Medicine, IRCCS Candiolo, Candiolo, Italy.*
[6] *INFN, Sezione di Torino, Torino, Italy.*

(Dated: February 11, 2026)

SUPPLEMENTARY MATERIAL

APPENDIX A1: DIRECT COUPLING ANALYSIS DETAILS

Summary Statistics

A Multiple Sequence Alignment $\mathcal{D}$ composed of M sequences of length L can be used to extract summary statistics representing the protein family. In particular, single- and pair-wise frequency counts of the amino acids in the alignment are defined as:

$$f_i(a) = \frac{1}{M}\sum_{m=1}^{M}\delta(a, a_i^m) \quad ,$$
$$f_{i,j}(a,b) = \frac{1}{M}\sum_{m=1}^{M}\delta(a, a_i^m)\delta(b, a_j^m) \quad . \tag{1}$$

The application of a maximum entropy principle to the frequency counts returns the Potts model at the base of every DCA method. Higher statistics would produce other terms in the Hamiltonian which could not be inferred due to the relatively small size of the datasets at hand [1, 2].

A good generative model should be able to reproduce the summary statistics and higher statistics derived from it, as in the case of the 2-site connected correlation:

$$c_{ij}(a,b) = f_{i,j}(a,b) - f_i(a)f_j(b) \quad . \tag{2}$$

Sequence re-weighting and effective number of sequences

Sequences in a natural MSA are not truly independently distributed. Indeed, a certain degree of homology arises due to the phylogenetic relationships between different sequences, bringing about probabilistic biases towards specific sets of sequences. To avoid the over-sampling of similar sequences and the relative under-representation of other sequences, it is necessary to introduce a reweighting scheme. For each sequence $\mathbf{a}^i$, a weight is defined as the inverse of the number of sequences similar to it:

$$w^\alpha = \frac{1}{m^\alpha} = \left|\left\{\beta \middle| 1 \le \beta \le M, \mathrm{seqid}(\mathbf{a}^\alpha, \mathbf{a}^\beta) > xL\right\}\right|^{-1} \quad , \tag{3}$$

* francesco.caredda@polito.it

where x is a similarity threshold that we set equal to 0.9. The effective number of sequences (effective depth) of the MSA M_{eff} is then defined as the sum of each weight:

$$M_{\text{eff}} = \sum_{i=1}^{M} \frac{1}{m^i} \quad . \tag{4}$$

Using this re-weighting scheme, the frequency counts can be written as:

$$f_i(a) = \frac{1}{M_{\text{eff}}} \sum_{m=1}^{M} w^m \delta(a, a_i^m) \quad ,$$
$$f_{i,j}(a,b) = \frac{1}{M_{\text{eff}}} \sum_{m=1}^{M} w^m \delta(a, a_i^m) \delta(b, a_j^m) \quad . \tag{5}$$

Depending on the effective depth of an MSA, a given DCA method can be more or less efficient, with different methods presenting different precision thresholds [2].

APPENDIX A2: MATHEMATICAL FOUNDATION OF THE FEATURE-CONDITIONED AUTOREGRESSIVE MODEL

Let us assume that we have an MSA of M one-hot encoded sequences of length L over an alphabet of q symbols ($q = 21$ for proteins). We can extract the rotation matrix $U \in \mathbb{R}^{d\times Lq}$, such that the application $U\mathbf{a} = \mathbf{y}$ is the projection of an amino-acid sequence $\mathbf{a}$ in the d-dimensional space spanned by the eigenvectors associated to the largest d eigenvalues of covariance matrix C^{emp}. Let us define the sequence

$$\mathbf{x}^\alpha := (\mathbf{a}_1^\alpha, \dots, \mathbf{a}_L^\alpha, y_1^\alpha, \dots, y_d^\alpha) := (\mathbf{a}^\alpha, \mathbf{y}^\alpha)$$

$$\begin{cases} \mathbf{a}^\alpha := (\mathbf{a}_1^\alpha, \dots, \mathbf{a}_L^\alpha) & \text{is an } Lq \text{ dimensional vector} \\ \mathbf{y}^\alpha = U\mathbf{a}^\alpha & \text{is a } d \text{ dimensional vector} \end{cases}$$

where $\mathbf{a}_i$ is the q-dimensional one-hot encoded representation of amino-acid a_i. We aim at learning the joint probability distribution $P(\mathbf{a}, \mathbf{y})$ from our data. We note that

$$P(\mathbf{a}, \mathbf{y}) = P(\mathbf{y}|\mathbf{a})P(\mathbf{a}) = \underbrace{\delta^{(d)}(\mathbf{y}-U\mathbf{a})}_{P(\mathbf{y}|\mathbf{a})}\frac{e^{-H(\mathbf{a})}}{Z} \tag{6}$$

where: (i) we assume in the last step that the prior distribution of $\mathbf{y}$ is uniform, $\delta^{(d)}(\mathbf{x}) := \prod_{i=1}^{d} \delta(x_i)$ is the d-dimensional Kronecker delta, and (ii) we model $P(\mathbf{a})$ as a Potts model defined by the energy function $H(\mathbf{a})$. The joint probability distribution $P(\mathbf{a}, \mathbf{y})$ is amenable to autoregressive representation:

$$P(\mathbf{a}, \mathbf{y}) = P(\mathbf{y})P(\mathbf{a}_1|\mathbf{y})P(\mathbf{a}_2|\mathbf{y}, \mathbf{a}_1) \cdots P(\mathbf{a}_L|\mathbf{y}, \mathbf{a}_1, \dots, \mathbf{a}_{L-1}) := P(\mathbf{y}) \prod_{i=1}^{L} P(\mathbf{a}_i|\mathbf{y}, \mathbf{a}_{<i})$$

where $\mathbf{a}_{<i} := \{a_1, \dots, a_{i-1}\}$ with the convention that $\mathbf{a}_{<1}$ is the empty set.

Gaussian Approximation

To proceed analytically, we make use of a Gaussian approximation for $P(\mathbf{a}) = \exp(-H)/Z$. Also, we relax the one-hot-encoded nature of the variables $\mathbf{a}_i$ to real values. In this case, we have that

$$P(\mathbf{a}) = \mathcal{N}(\mathbf{a}|\boldsymbol{\mu}, \Sigma) = \frac{1}{\sqrt{(2\pi)^{Lq} \det \Sigma}} e^{-\frac{1}{2}(\mathbf{a}-\boldsymbol{\mu})^\dagger \Sigma^{-1} (\mathbf{a}-\boldsymbol{\mu})^\dagger}$$

Eq. 6 becomes:

$$P(\mathbf{a}, \mathbf{y}) = P(\mathbf{y}|\mathbf{a})P(\mathbf{a}) = \delta^{(d)}(\mathbf{y}-U\mathbf{a})\mathcal{N}(\mathbf{a}|\boldsymbol{\mu}, \Sigma)$$

We can compute the marginal $P(\mathbf{y})$ which is a Gaussian

$$P(\mathbf{y}) = \int d\mathbf{a} P(\mathbf{a}, \mathbf{y}) = \mathcal{N}\left(\mathbf{y}\middle|U\boldsymbol{\mu}, U\Sigma U^\dagger\right)$$

Computation of the marginal $P(\boldsymbol{y})$

Let us consider the case $P(\mathbf{y}|\mathbf{a}) = \delta^{(d)}(\mathbf{y}{-}U\mathbf{a})$. We have that

$$
\begin{aligned}
P(\mathbf{y}) &= \frac{1}{\sqrt{(2\pi)^{Lq}\det(\Sigma)}}\int d\mathbf{a} e^{-\frac{1}{2}(\mathbf{a}-\boldsymbol{\mu})^\dagger\Sigma^{-1}(\mathbf{a}-\boldsymbol{\mu})}\delta^{(d)}(\mathbf{y}{-}U\mathbf{a}) \\
&= \frac{1}{\sqrt{(2\pi)^{Lq}\det(\Sigma)}}\int d\mathbf{a}\frac{d\mathbf{k}}{(2\pi)^d} e^{-\frac{1}{2}(\mathbf{a}-\boldsymbol{\mu})^\dagger\Sigma^{-1}(\mathbf{a}-\boldsymbol{\mu}))} e^{i\mathbf{k}^\dagger(\mathbf{y}-U\mathbf{a})} \\
&= \frac{1}{\sqrt{(2\pi)^{Lq}\det(\Sigma)}}\int \frac{d\mathbf{k}}{(2\pi)^d}\int d\mathbf{a} e^{-\frac{1}{2}(\mathbf{a}-\boldsymbol{\mu})^\dagger\Sigma^{-1}(\mathbf{a}-\boldsymbol{\mu}))+i\mathbf{k}^\dagger(\mathbf{y}-U\mathbf{a})} \\
&= \frac{1}{\sqrt{(2\pi)^{Lq}\det(\Sigma)}}\int \frac{d\mathbf{k}}{(2\pi)^d}\int d\mathbf{a} e^{-\frac{1}{2}\mathbf{a}^\dagger\Sigma^{-1}\mathbf{a}+\boldsymbol{\mu}^\dagger\Sigma^{-1}\mathbf{a}-\frac{1}{2}\boldsymbol{\mu}^\dagger\Sigma^{-1}\boldsymbol{\mu}+i\mathbf{k}^\dagger\mathbf{y}-i\mathbf{k}^\dagger U\mathbf{a}} \\
&= \frac{1}{\sqrt{(2\pi)^{Lq}\det(\Sigma)}}\int \frac{d\mathbf{k}}{(2\pi)^d} e^{-\frac{1}{2}\boldsymbol{\mu}^\dagger\Sigma^{-1}\boldsymbol{\mu}+i\mathbf{k}^\dagger\mathbf{y}}\int d\mathbf{a} e^{-\frac{1}{2}\mathbf{a}^\dagger\Sigma^{-1}\mathbf{a}+(\boldsymbol{\mu}^\dagger\Sigma^{-1}-i\mathbf{k}^\dagger U)\mathbf{a}} \\
&= \frac{1}{\sqrt{(2\pi)^{Lq}\det(\Sigma)}}\int \frac{d\mathbf{k}}{(2\pi)^d} e^{-\frac{1}{2}\boldsymbol{\mu}^\dagger\Sigma^{-1}\boldsymbol{\mu}+i\mathbf{k}^\dagger\mathbf{y}}\int d\mathbf{a} e^{-\frac{1}{2}\mathbf{a}^\dagger\Sigma^{-1}\mathbf{a}+(\Sigma^{-1}\boldsymbol{\mu}-iU^\dagger\mathbf{k})^\dagger\mathbf{a}} \\
&= \frac{\sqrt{(2\pi)^{Lq}\det(\Sigma)}}{\sqrt{(2\pi)^{Lq}\det(\Sigma)}}\int \frac{d\mathbf{k}}{(2\pi)^d} e^{-\frac{1}{2}\boldsymbol{\mu}^\dagger\Sigma^{-1}\boldsymbol{\mu}+i\mathbf{k}^\dagger\mathbf{y}+\frac{1}{2}(\Sigma^{-1}\boldsymbol{\mu}-iU^\dagger\mathbf{k})^\dagger\Sigma(\Sigma^{-1}\boldsymbol{\mu}-iU^\dagger\mathbf{k})} \\
&= \underbrace{e^{-\frac{1}{2}\boldsymbol{\mu}^\dagger\Sigma^{-1}\boldsymbol{\mu}+\frac{1}{2}(\Sigma^{-1}\boldsymbol{\mu})^\dagger\Sigma(\Sigma^{-1}\boldsymbol{\mu})}}_{=1}\int \frac{d\mathbf{k}}{(2\pi)^d} e^{i\mathbf{k}^\dagger\mathbf{y}-\frac{1}{2}(U^\dagger\mathbf{k})^\dagger\Sigma U^\dagger\mathbf{k}-i(\Sigma^{-1}\boldsymbol{\mu})^\dagger\Sigma U^\dagger\mathbf{k}} \\
&= \int \frac{d\mathbf{k}}{(2\pi)^d} e^{-\frac{1}{2}(U^\dagger\mathbf{k})^\dagger\Sigma U^\dagger\mathbf{k}+i\left(\mathbf{y}^\dagger-\boldsymbol{\mu}^\dagger\Sigma^{-1}\Sigma U^\dagger\right)\mathbf{k}} \\
&= \int \frac{d\mathbf{k}}{(2\pi)^d} e^{-\frac{1}{2}\mathbf{k}^\dagger U\Sigma U^\dagger\mathbf{k}+i(\mathbf{y}-U\boldsymbol{\mu})^\dagger\mathbf{k}} \\
&= \frac{\sqrt{(2\pi)^d}}{(2\pi)^d\sqrt{\det(U\Sigma U^\dagger)}} e^{-\frac{1}{2}(\mathbf{y}-U\boldsymbol{\mu})^\dagger\left(U\Sigma U^\dagger\right)^{-1}(\mathbf{y}-U\boldsymbol{\mu})} \\
&= \frac{1}{\sqrt{(2\pi)^d\det(U\Sigma U^\dagger)}} e^{-\frac{1}{2}(\mathbf{y}-U\boldsymbol{\mu})^\dagger\left(U\Sigma U^\dagger\right)^{-1}(\mathbf{y}-U\boldsymbol{\mu})} \\
&= \mathcal{N}\left(\mathbf{y}\middle|U\boldsymbol{\mu}, U\Sigma U^\dagger\right)
\end{aligned}
$$

Alternatively, we could have computed the same result by observing that $P(\mathbf{y})$ is Gaussian and its first two moments can be computed as:

$$
\begin{aligned}
\langle\mathbf{y}\rangle_{P(\mathbf{y})} &= \int d\mathbf{a}P(\mathbf{a})\int d\mathbf{y}\delta^{(d)}(\mathbf{y}{-}U\mathbf{a})\mathbf{y} = U\boldsymbol{\mu} \\
\left\langle\left(\mathbf{y}-\langle\mathbf{y}\rangle_{P(\mathbf{y})}\right)\left(\mathbf{y}-\langle\mathbf{y}\rangle_{P(\mathbf{y})}\right)^\dagger\right\rangle_{P(\mathbf{y})} &= \int d\mathbf{a}P(\mathbf{a})\int d\mathbf{y}\delta^{(d)}(\mathbf{y}{-}U\mathbf{a})\left(\mathbf{y}-\langle\mathbf{y}\rangle_{P(\mathbf{y})}\right)\left(\mathbf{y}-\langle\mathbf{y}\rangle_{P(\mathbf{y})}\right)^\dagger \\
&= U\Sigma U^\dagger
\end{aligned}
$$

In the following, we will use this technique extensively.

So far, we can write the conditioned distribution $P(\mathbf{a}|\mathbf{y})$ as

$$P(\mathbf{a}|\mathbf{y}) = \frac{P(\mathbf{a},\mathbf{y})}{P(\mathbf{y})} = \frac{\overbrace{\delta^{(d)}(\mathbf{y}-U\mathbf{a})}^{P(\mathbf{y}|\mathbf{a})}\overbrace{\mathcal{N}(\mathbf{a}|\boldsymbol{\mu},\Sigma)}^{P(\mathbf{a})}}{\underbrace{\mathcal{N}\left(\mathbf{y}\middle|U\boldsymbol{\mu},U\Sigma U^\dagger\right)}_{P(\mathbf{y})}}$$

$$= \sqrt{\frac{(2\pi)^d\det(U\Sigma U^\dagger)}{(2\pi)^{Lq}\det(\Sigma)}}e^{-\frac{1}{2}(\mathbf{a}-\boldsymbol{\mu})^\dagger\Sigma^{-1}(\mathbf{a}-\boldsymbol{\mu})+\frac{1}{2}(\mathbf{y}-U\boldsymbol{\mu})^\dagger\left(U\Sigma U^\dagger\right)^{-1}(\mathbf{y}-U\boldsymbol{\mu})}\delta^{(d)}(\mathbf{y}-U\mathbf{a})$$

$$\propto e^{-\frac{1}{2}\mathbf{a}^\dagger\Sigma^{-1}\mathbf{a}+\boldsymbol{\mu}^\dagger\Sigma^{-1}\mathbf{a}+\frac{1}{2}\mathbf{y}^\dagger U\Sigma^{-1}U^\dagger\mathbf{y}+\boldsymbol{\mu}^\dagger\Sigma^{-1}U^\dagger\mathbf{y}}\delta^{(d)}(\mathbf{y}-U\mathbf{a})$$

so that

$$P(\mathbf{a}|\mathbf{y}) = Ke^{-\frac{1}{2}\mathbf{a}^\dagger\Sigma^{-1}\mathbf{a}+\boldsymbol{\mu}^\dagger\Sigma^{-1}\mathbf{a}+\frac{1}{2}\mathbf{y}^\dagger U\Sigma^{-1}U^\dagger\mathbf{y}+\boldsymbol{\mu}^\dagger\Sigma^{-1}U^\dagger\mathbf{y}}\delta^{(d)}(\mathbf{y}-U\mathbf{a})$$

where K is a normalization constant that does not affect the following computations.

First marginal The first marginal of the conditioned distribution $P(\mathbf{a}|\mathbf{y})$ can be computed as

$$\begin{aligned}
P(\mathbf{a}_1|\mathbf{y}) &= Ke^{\frac{1}{2}\mathbf{y}^\dagger U\Sigma^{-1}U^\dagger\mathbf{y}+\boldsymbol{\mu}^\dagger\Sigma^{-1}U^\dagger\mathbf{y}}\int d\mathbf{a}_{-1}e^{-\frac{1}{2}\mathbf{a}^\dagger\Sigma^{-1}\mathbf{a}+\boldsymbol{\mu}^\dagger\Sigma^{-1}\mathbf{a}}\int\frac{d\mathbf{k}}{(2\pi)^d}e^{i\mathbf{k}^\dagger(\mathbf{y}-U\mathbf{a})}\\
&= Ke^{\frac{1}{2}\mathbf{y}^\dagger U\Sigma^{-1}U^\dagger\mathbf{y}+\boldsymbol{\mu}^\dagger\Sigma^{-1}U^\dagger\mathbf{y}-\frac{1}{2}\mathbf{a}_1^\dagger\left(\Sigma^{-1}\right)_1\mathbf{a}_1+\boldsymbol{\mu}_1^\dagger\left(\Sigma^{-1}\right)_1\mathbf{a}_1}\int d\mathbf{a}_{-1}e^{-\frac{1}{2}\mathbf{a}_{-1}^\dagger\left(\Sigma^{-1}\right)_{-1}\mathbf{a}_{-1}+\boldsymbol{\mu}_{-1}^\dagger\left(\Sigma^{-1}\right)_{-1}\mathbf{a}_{-1}}\times\\
&\times\int\frac{d\mathbf{k}}{(2\pi)^d}e^{i\mathbf{k}^\dagger(\mathbf{y}-U_1\mathbf{a}_1-U_{-1}\mathbf{a}_{-1})} =\\
&= Ke^{\frac{1}{2}\mathbf{y}^\dagger U\Sigma^{-1}U^\dagger\mathbf{y}+\boldsymbol{\mu}^\dagger\Sigma^{-1}U^\dagger\mathbf{y}-\frac{1}{2}\mathbf{a}_1^\dagger\left(\Sigma^{-1}\right)_1\mathbf{a}_1+\boldsymbol{\mu}_1^\dagger\left(\Sigma^{-1}\right)_1\mathbf{a}_1}\times\\
&\times\int\frac{d\mathbf{k}}{(2\pi)^d}e^{i\left(\mathbf{y}^\dagger-\mathbf{a}_1^\dagger U_1^\dagger\right)\mathbf{k}}\int d\mathbf{a}_{-1}e^{-\frac{1}{2}\mathbf{a}_{-1}^\dagger\left(\Sigma^{-1}\right)_{-1}\mathbf{a}_{-1}+\left(\left(\Sigma^{-1}\right)_{-1}\boldsymbol{\mu}_{-1}-iU_{-1}^\dagger\mathbf{k}\right)^\dagger\mathbf{a}_{-1}} =\\
&= Ke^{\frac{1}{2}\mathbf{y}^\dagger U\Sigma^{-1}U^\dagger\mathbf{y}+\boldsymbol{\mu}^\dagger\Sigma^{-1}U^\dagger\mathbf{y}-\frac{1}{2}\mathbf{a}_1^\dagger\left(\Sigma^{-1}\right)_1\mathbf{a}_1+\boldsymbol{\mu}_1^\dagger\left(\Sigma^{-1}\right)_1\mathbf{a}_1}\times\\
&\times\int\frac{d\mathbf{k}}{(2\pi)^d}e^{i\left(\mathbf{y}^\dagger-\mathbf{a}_1^\dagger U_1^\dagger\right)\mathbf{k}+\frac{1}{2}\left(\left(\Sigma^{-1}\right)_{-1}\boldsymbol{\mu}_{-1}-iU_{-1}^\dagger\mathbf{k}\right)^\dagger(\Sigma)_{-1}\left(\left(\Sigma^{-1}\right)_{-1}\boldsymbol{\mu}_{-1}-iU_{-1}^\dagger\mathbf{k}\right)} =\\
&= Ke^{\frac{1}{2}\mathbf{y}^\dagger U\Sigma^{-1}U^\dagger\mathbf{y}+\boldsymbol{\mu}^\dagger\Sigma^{-1}U^\dagger\mathbf{y}-\frac{1}{2}\mathbf{a}_1^\dagger\left(\Sigma^{-1}\right)_1\mathbf{a}_1+\boldsymbol{\mu}_1^\dagger\left(\Sigma^{-1}\right)_1\mathbf{a}_1}\times\\
&\times\int\frac{d\mathbf{k}}{(2\pi)^d}e^{i\left(\mathbf{y}^\dagger-\mathbf{a}_1^\dagger U_1^\dagger\right)\mathbf{k}+\frac{1}{2}\boldsymbol{\mu}_{-1}^\dagger\left(\Sigma^{-1}\right)_{-1}\boldsymbol{\mu}_{-1}-\frac{1}{2}\mathbf{k}^\dagger U_{-1}(\Sigma)_{-1}U_{-1}^\dagger\mathbf{k}-i\boldsymbol{\mu}_{-1}U_{-1}^\dagger\mathbf{k}} =\\
&= Ke^{\frac{1}{2}\mathbf{y}^\dagger U\Sigma^{-1}U^\dagger\mathbf{y}+\boldsymbol{\mu}^\dagger\Sigma^{-1}U^\dagger\mathbf{y}-\frac{1}{2}\mathbf{a}_1^\dagger\left(\Sigma^{-1}\right)_1\mathbf{a}_1+\boldsymbol{\mu}_1^\dagger\left(\Sigma^{-1}\right)_1\mathbf{a}_1}\int\frac{d\mathbf{k}}{(2\pi)^d}e^{-\frac{1}{2}\mathbf{k}^\dagger U_{-1}(\Sigma)_{-1}U_{-1}^\dagger\mathbf{k}+i(\mathbf{y}-U_{-1}\boldsymbol{\mu}_{-1}-U_1\mathbf{a}_1)^\dagger\mathbf{k}}\\
&= Ke^{\frac{1}{2}\mathbf{y}^\dagger U\Sigma^{-1}U^\dagger\mathbf{y}+\boldsymbol{\mu}^\dagger\Sigma^{-1}U^\dagger\mathbf{y}-\frac{1}{2}\mathbf{a}_1^\dagger\left(\Sigma^{-1}\right)_1\mathbf{a}_1+\boldsymbol{\mu}_1^\dagger\left(\Sigma^{-1}\right)_1\mathbf{a}_1-\frac{1}{2}(\mathbf{y}-U_{-1}\boldsymbol{\mu}_{-1}-U_1\mathbf{a}_1)^\dagger\left(U_{-1}(\Sigma)_{-1}U_{-1}^\dagger\right)^{-1}(\mathbf{y}-U_{-1}\boldsymbol{\mu}_{-1}-U_1\mathbf{a}_1)}\\
&= Ke^{\frac{1}{2}\mathbf{y}^\dagger U\Sigma_a^{-1}U^\dagger\mathbf{y}+\boldsymbol{\mu}^\dagger\Sigma^{-1}U^\dagger\mathbf{y}-\frac{1}{2}\mathbf{a}_1^\dagger\left(\Sigma^{-1}\right)_1\mathbf{a}_1+\boldsymbol{\mu}_1^\dagger\left(\Sigma^{-1}\right)_1\mathbf{a}_1-\frac{1}{2}(\mathbf{y}-U_{-1}\boldsymbol{\mu}_{-1}-U_1\mathbf{a}_1)^\dagger U_{-1}\left(\Sigma^{-1}\right)_{-1}U_{-1}^\dagger(\mathbf{y}-U_{-1}\boldsymbol{\mu}_{-1}-U_1\mathbf{a}_1)}
\end{aligned}$$

The marginal distribution $P(\boldsymbol{a}_1|\boldsymbol{y})$ remains a Gaussian distribution that can be fully characterized by its mean and variance. The mean can be computed as the maximum of the argument of the exponential, while the variance as the opposite of the inverse of its second derivative with respect to the variable $\boldsymbol{a}_1$:

$$\begin{aligned}
\boldsymbol{\mu}_{\boldsymbol{a}_1|\boldsymbol{y}} := \boldsymbol{a}_1^* = \arg\max_{\boldsymbol{a}_1}\Big[&\frac{1}{2}\mathbf{y}^\dagger U\Sigma_a^{-1}U^\dagger\mathbf{y}+\boldsymbol{\mu}^\dagger\Sigma^{-1}U^\dagger\mathbf{y}-\frac{1}{2}\mathbf{a}_1^\dagger\left(\Sigma^{-1}\right)_1\mathbf{a}_1+\\
&+\boldsymbol{\mu}_1^\dagger\left(\Sigma^{-1}\right)_1\mathbf{a}_1-\frac{1}{2}\left(\mathbf{y}-U_{-1}\boldsymbol{\mu}_{-1}-U_1\mathbf{a}_1\right)^\dagger U_{-1}\left(\Sigma^{-1}\right)_{-1}U_{-1}^\dagger\left(\mathbf{y}-U_{-1}\boldsymbol{\mu}_{-1}-U_1\mathbf{a}_1\right)\Big]
\end{aligned}$$

$$\Sigma^{-1}_{\boldsymbol{a}_1|\boldsymbol{y}} := -\frac{d^2}{d\boldsymbol{a}_1^2}\Big[\frac{1}{2}\mathbf{y}^\dagger U \Sigma_a^{-1} U^\dagger \mathbf{y} + \boldsymbol{\mu}^\dagger \Sigma^{-1} U^\dagger \mathbf{y} - \frac{1}{2}\mathbf{a}_1^\dagger \left(\Sigma^{-1}\right)_1 \mathbf{a}_1 + \\ + \boldsymbol{\mu}_1^\dagger \left(\Sigma^{-1}\right)_1 \mathbf{a}_1 - \frac{1}{2}\left(\mathbf{y} - U_{-1}\boldsymbol{\mu}_{-1} - U_1\mathbf{a}_1\right)^\dagger U_{-1}\left(\Sigma^{-1}\right)_{-1} U_{-1}^\dagger \left(\mathbf{y} - U_{-1}\boldsymbol{\mu}_{-1} - U_1\mathbf{a}_1\right)\Big]$$

Recalling the identities:

$$\frac{d\boldsymbol{x}^T\boldsymbol{a}}{d\boldsymbol{x}} = \frac{d\boldsymbol{a}^T\boldsymbol{x}}{d\boldsymbol{x}} = \boldsymbol{a}$$

$$\frac{d\left(\boldsymbol{B}\boldsymbol{x}+\boldsymbol{b}\right)^T \boldsymbol{C}\left(\boldsymbol{D}\boldsymbol{x}+\boldsymbol{d}\right)}{d\boldsymbol{x}} = \boldsymbol{B}^T\boldsymbol{C}\left(\boldsymbol{D}\boldsymbol{x}+\boldsymbol{d}\right) + \boldsymbol{D}^T\boldsymbol{C}^T\left(\boldsymbol{B}\boldsymbol{x}+\boldsymbol{b}\right)$$

we have that the mean is given by:

$$\frac{d}{d\boldsymbol{a_1}}\Big[\frac{1}{2}\mathbf{y}^\dagger U \Sigma^{-1} U^\dagger \mathbf{y} + \boldsymbol{\mu}^\dagger \Sigma^{-1} U^\dagger \mathbf{y} - \frac{1}{2}\mathbf{a}_1^\dagger \left(\Sigma^{-1}\right)_1 \mathbf{a}_1 + \\ + \boldsymbol{\mu}_1^\dagger \left(\Sigma^{-1}\right)_1 \mathbf{a}_1 - \frac{1}{2}\left(\mathbf{y} - U_{-1}\boldsymbol{\mu}_{-1} - U_1\mathbf{a}_1\right)^\dagger U_{-1}\left(\Sigma_a^{-1}\right)_{-1} U_{-1}^\dagger \left(\mathbf{y} - U_{-1}\boldsymbol{\mu}_{-1} - U_1\mathbf{a}_1\right)\Big] = \\ \frac{d}{d\boldsymbol{a_1}}\left[-\frac{1}{2}\mathbf{a}_1^\dagger \left(\Sigma^{-1}\right)_1 \mathbf{a}_1 + \boldsymbol{\mu}_1^\dagger \left(\Sigma^{-1}\right)_1 \mathbf{a}_1 - \frac{1}{2}\left(\mathbf{y} - U_{-1}\boldsymbol{\mu}_{-1} - U_1\mathbf{a}_1\right)^\dagger U_{-1}\left(\Sigma^{-1}\right)_{-1} U_{-1}^\dagger \left(\mathbf{y} - U_{-1}\boldsymbol{\mu}_{-1} - U_1\mathbf{a}_1\right)\right] = \\ -\left(\Sigma^{-1}\right)_1 \mathbf{a}_1 + \left(\Sigma^{-1}\right)_1 \boldsymbol{\mu}_1 - \left(U_1\right)^T U_{-1}\left(\Sigma^{-1}\right)_{-1} U_{-1}^\dagger \left(\mathbf{y} - U_{-1}\boldsymbol{\mu}_{-1} - U_1\mathbf{a}_1\right) = 0$$

$$\implies \quad \left(\Sigma^{-1}\right)_1 \mathbf{a}_1 - \left(U_1\right)^\dagger U_{-1}\left(\Sigma^{-1}\right)_{-1} U_{-1}^\dagger U_1 \mathbf{a}_1 = \left(\Sigma^{-1}\right)_1 \boldsymbol{\mu}_1 - \left(U_1\right)^\dagger U_{-1}\left(\Sigma^{-1}\right)_{-1} U_{-1}^\dagger \left(\mathbf{y} - U_{-1}\boldsymbol{\mu}_{-1}\right)$$

$$\boldsymbol{\mu}^*_{\boldsymbol{a}_1|\boldsymbol{y}} := \boldsymbol{a}_1^* = \left[\left(\Sigma^{-1}\right)_1 - \left(U_1\right)^\dagger U_{-1}\left(\Sigma^{-1}\right)_{-1} U_{-1}^\dagger U_1\right]^{-1}\left[\left(\Sigma^{-1}\right)_1 \boldsymbol{\mu}_1 - \left(U_1\right)^\dagger U_{-1}\left(\Sigma^{-1}\right)_{-1} U_{-1}^\dagger \left(\mathbf{y} - U_{-1}\boldsymbol{\mu}_{-1}\right)\right] \\ = \Sigma_{\boldsymbol{a}_1|\boldsymbol{y}}\left[\left(\Sigma^{-1}\right)_1 \boldsymbol{\mu}_1 - \left(U_1\right)^\dagger U_{-1}\left(\Sigma^{-1}\right)_{-1} U_{-1}^\dagger \left(\mathbf{y} - U_{-1}\boldsymbol{\mu}_{-1}\right)\right] \qquad \in \mathbb{R}^{q,1}$$

while the variance is:

$$\Sigma^{-1}_{\boldsymbol{a}_1|\boldsymbol{y}} := -\frac{d^2}{d\boldsymbol{a_1^2}}\Big[\frac{1}{2}\mathbf{y}^\dagger U \Sigma^{-1} U^\dagger \mathbf{y} + \boldsymbol{\mu}^\dagger \Sigma^{-1} U^\dagger \mathbf{y} - \frac{1}{2}\mathbf{a}_1^\dagger \left(\Sigma^{-1}\right)_1 \mathbf{a}_1 + \\ + \boldsymbol{\mu}_1^\dagger \left(\Sigma^{-1}\right)_1 \mathbf{a}_1 - \frac{1}{2}\left(\mathbf{y} - U_{-1}\boldsymbol{\mu}_{-1} - U_1\mathbf{a}_1\right)^\dagger U_{-1}\left(\Sigma^{-1}\right)_{-1} U_{-1}^\dagger \left(\mathbf{y} - U_{-1}\boldsymbol{\mu}_{-1} - U_1\mathbf{a}_1\right)\Big] \\ = -\frac{d^2}{d\boldsymbol{a_1^2}}\left[-\frac{1}{2}\mathbf{a}_1^\dagger \left(\Sigma^{-1}\right)_1 \mathbf{a}_1 - \frac{1}{2}\left(\mathbf{y} - U_{-1}\boldsymbol{\mu}_{-1} - U_1\mathbf{a}_1\right)^\dagger U_{-1}\left(\Sigma^{-1}\right)_{-1} U_{-1}^\dagger \left(\mathbf{y} - U_{-1}\boldsymbol{\mu}_{-1} - U_1\mathbf{a}_1\right)\right] \\ = -\frac{d}{d\boldsymbol{a_1}}\left[-\left(\Sigma_a^{-1}\right)_1 \mathbf{a}_1 - \left(U_1\right)^\dagger U_{-1}\left(\Sigma^{\cdot 1}\right)_{-1} U_{-1}^\dagger \left(\mathbf{y} - U_{-1}\boldsymbol{\mu}_{-1} - U_1\mathbf{a}_1\right)\right] \\ = \left(\Sigma^{-1}\right)_1^\dagger - \left(\left(U_1\right)^\dagger U_{-1}\left(\Sigma^{-1}\right)_{-1} U_{-1}^\dagger U_1\right)^\dagger \\ = \left(\Sigma^{-1}\right)_1 - \left(U_1^\dagger U_{-1}\left(\Sigma^{-1}\right)_{-1} U_{-1}^\dagger \left(U_1\right)\right) \qquad \in \mathbb{R}^{q,q}$$

So the marginal can be written as

$$P(\mathbf{a}_1|\mathbf{y}) = \frac{1}{\sqrt{(2\pi)^q \det(\Sigma_{1|y})}} e^{-\frac{1}{2}\left(\boldsymbol{a}_1 - \boldsymbol{\mu}_{\boldsymbol{a}_1|\boldsymbol{y}}\right)^T \Sigma^{-1}_{\boldsymbol{a}_1|\boldsymbol{y}}\left(\boldsymbol{a}_1 - \boldsymbol{\mu}_{\boldsymbol{a}_1|\boldsymbol{y}}\right)^T} = \frac{e^{\mu^T_{\boldsymbol{a}_1|\boldsymbol{y}}\Sigma^{-1}_{\boldsymbol{a}_1|\boldsymbol{y}}\mu_{\boldsymbol{a}_1|\boldsymbol{y}}}}{\sqrt{(2\pi)^q \det(\Sigma_{\boldsymbol{a}_1|\boldsymbol{y}})}} e^{-\frac{1}{2}\boldsymbol{a}_1^T\Sigma^{-1}_{\boldsymbol{a}_1|\boldsymbol{y}}\boldsymbol{a}_1 + \boldsymbol{a}_1^T\Sigma^{-1}_{\boldsymbol{a}_1|\boldsymbol{y}}\mu_{\boldsymbol{a}_1|\boldsymbol{y}}} \\ = \frac{e^{\mu^T_{\boldsymbol{a}_1|\boldsymbol{y}}\Sigma^{-1}_{\boldsymbol{a}_1|\boldsymbol{y}}\mu_{\boldsymbol{a}_1|\boldsymbol{y}}}}{\sqrt{(2\pi)^q \det(\Sigma_{\boldsymbol{a}_1|\boldsymbol{y}})}} e^{-\frac{1}{2}\boldsymbol{a}_1^T\Sigma^{-1}_{\boldsymbol{a}_1|\boldsymbol{y}}\boldsymbol{a}_1 + \boldsymbol{a}_1^T\left[\left(\Sigma^{-1}\right)_1\boldsymbol{\mu}_1 - \left(U_1\right)^\dagger U_{-1}\left(\Sigma^{-1}\right)_{-1} U_{-1}^\dagger\left(\mathbf{y} - U_{-1}\boldsymbol{\mu}_{-1}\right)\right]}$$

In this context the notation $\left(\Sigma^{-1}\right)_1 \in \mathbb{R}^{q,q}$ refers to the first block of matrix Σ^{-1}, while $\left(\Sigma^{-1}\right)_{-1} \in \mathbb{R}^{q(L-1),q(L-1)}$ refers to the sub-matrix defined by removing the blocks relative to the first amino-acid. The same goes for $U_1 \in \mathbb{R}^{d,q}$ and $U_{-1} \in \mathbb{R}^{d,q(L-1)}$.

k-th marginal Generalizing the previous computation, we can compute the k-th marginal of the distribution $P(\boldsymbol{a}_k|\boldsymbol{a}_{<k},\boldsymbol{y})$ conditioned to all previous positions and to PCA components $\boldsymbol{y}$. Using Bayes' rule we can write:

$$
\begin{aligned}
P(\boldsymbol{a}|\boldsymbol{y}) &= \frac{P(\boldsymbol{a},\boldsymbol{y})}{P(\boldsymbol{y})} \\
&= \frac{P(\boldsymbol{a}_k,\dots,\boldsymbol{a}_L|\boldsymbol{a}_1,\dots\boldsymbol{a}_{k-1},\boldsymbol{y})P(\boldsymbol{a}_1,\dots\boldsymbol{a}_{k-1},\boldsymbol{y})}{P(\boldsymbol{y})} \\
&= \frac{P(\boldsymbol{a}_k,\dots,\boldsymbol{a}_L|\boldsymbol{a}_1,\dots\boldsymbol{a}_{k-1},\boldsymbol{y})\prod_{i=1}^{k-1}P(\boldsymbol{a}_i|\boldsymbol{a}_{<i},\boldsymbol{y})P(\boldsymbol{y})}{P(\boldsymbol{y})} \\
&= P(\boldsymbol{a}_k,\dots,\boldsymbol{a}_L|\boldsymbol{a}_1,\dots\boldsymbol{a}_{k-1},\boldsymbol{y})\prod_{i=1}^{k-1}P(\boldsymbol{a}_i|\boldsymbol{a}_{<i},\boldsymbol{y})
\end{aligned}
$$

$$
P(\boldsymbol{a}_k,\dots,\boldsymbol{a}_L|\boldsymbol{a}_1,\dots\boldsymbol{a}_{k-1},\boldsymbol{y}) = \frac{P(\boldsymbol{a}|\boldsymbol{y})}{\prod_{i=1}^{k-1}P(\boldsymbol{a}_i|\boldsymbol{a}_{<i},\boldsymbol{y})}
$$

From this, we can define the marginal as:

$$
P(\boldsymbol{a}_k|\boldsymbol{a}_1,\dots\boldsymbol{a}_{k-1},\boldsymbol{y}) = \int d\boldsymbol{a}_{>k}P(\boldsymbol{a}_k,\dots,\boldsymbol{a}_L|\boldsymbol{a}_1,\dots\boldsymbol{a}_{k-1},\boldsymbol{y}) = \int d\boldsymbol{a}_{>k}\frac{P(\boldsymbol{a}|\boldsymbol{y})}{\prod_{i=1}^{k-1}P(\boldsymbol{a}_i|\boldsymbol{a}_{<i},\boldsymbol{y})}
$$

The actual computation is quite straightforward

$$
\begin{aligned}
P(\boldsymbol{a}_k|\boldsymbol{a}_1,\dots\boldsymbol{a}_{k-1},\boldsymbol{y}) &= \int d\boldsymbol{a}_{>k}P(\boldsymbol{a}_k,\dots,\boldsymbol{a}_L|\boldsymbol{a}_1,\dots\boldsymbol{a}_{k-1},\boldsymbol{y}) = \int d\boldsymbol{a}_{>k}\frac{P(\boldsymbol{a}|\boldsymbol{y})}{\prod_{i=1}^{k-1}P(\boldsymbol{a}_i|\boldsymbol{a}_{<i},\boldsymbol{y})} \\
&= \frac{1}{\prod_{i=1}^{k-1}P(\boldsymbol{a}_i|\boldsymbol{a}_{<i},\boldsymbol{y})}\int d\boldsymbol{a}_{>k}P(\boldsymbol{a}|\boldsymbol{y}) = \\
&\propto \int d\boldsymbol{a}_{>k}e^{-\frac{1}{2}\mathbf{a}^\dagger\Sigma^{-1}\mathbf{a}+\boldsymbol{\mu}^\dagger\Sigma^{-1}\mathbf{a}+\frac{1}{2}\mathbf{y}^\dagger U\Sigma^{-1}U^\dagger\mathbf{y}+\boldsymbol{\mu}^\dagger\Sigma^{-1}U^\dagger\mathbf{y}}\delta^{(d)}(\mathbf{y}-U\mathbf{a}) \\
&\propto e^{-\frac{1}{2}\mathbf{a}^\dagger_{\le k}\Sigma^{-1}_{\le k}\mathbf{a}_{\le k}+\boldsymbol{\mu}^\dagger_{\le k}\Sigma^{-1}_{\le k}\mathbf{a}_{\le k}}\int d\boldsymbol{a}_{>k}e^{-\frac{1}{2}\mathbf{a}^\dagger_{>k}\Sigma^{-1}_{>k}\mathbf{a}_{>k}+\boldsymbol{\mu}^\dagger_{>k}\Sigma^{-1}_{>k}\mathbf{a}_{>k}}\int\frac{d\boldsymbol{k}}{(2\pi)^d}e^{i\boldsymbol{k}^\dagger(\boldsymbol{y}-U\boldsymbol{a})} \\
&\propto e^{-\frac{1}{2}\mathbf{a}^\dagger_{\le k}\Sigma^{-1}_{\le k}\mathbf{a}_{\le k}+\boldsymbol{\mu}^\dagger_{\le k}\Sigma^{-1}_{\le k}\mathbf{a}_{\le k}}\int\frac{d\boldsymbol{k}}{(2\pi)^d}e^{i\boldsymbol{k}^\dagger(\boldsymbol{y}-U_{\le k}\boldsymbol{a}_{\le k})}\int d\boldsymbol{a}_{>k}e^{-\frac{1}{2}\mathbf{a}^\dagger_{>k}\Sigma^{-1}_{>k}\mathbf{a}_{>k}+\left(\Sigma^{-1\dagger}_{>k}\boldsymbol{\mu}_{>k}-iU^\dagger_{>k}\boldsymbol{k}\right)^\dagger\boldsymbol{a}_{>k}} \\
&\propto e^{-\frac{1}{2}\mathbf{a}^\dagger_{\le k}\Sigma^{-1}_{\le k}\mathbf{a}_{\le k}+\boldsymbol{\mu}^\dagger_{\le k}\Sigma^{-1}_{\le k}\mathbf{a}_{\le k}}\int\frac{d\boldsymbol{k}}{(2\pi)^d}e^{i\boldsymbol{k}^\dagger(\boldsymbol{y}-U_{\le k}\boldsymbol{a}_{\le k})+\frac{1}{2}\left(\Sigma^{-1\dagger}_{>k}\boldsymbol{\mu}_{>k}-iU^\dagger_{>k}\boldsymbol{k}\right)^\dagger\Sigma_{>k}\left(\Sigma^{-1\dagger}_{>k}\boldsymbol{\mu}_{>k}-iU^\dagger_{>k}\boldsymbol{k}\right)} \\
&\propto e^{-\frac{1}{2}\mathbf{a}^\dagger_{\le k}\Sigma^{-1}_{\le k}\mathbf{a}_{\le k}+\boldsymbol{\mu}^\dagger_{\le k}\Sigma^{-1}_{\le k}\mathbf{a}_{\le k}}\int\frac{d\boldsymbol{k}}{(2\pi)^d}e^{i\boldsymbol{k}^\dagger(\boldsymbol{y}-U_{\le k}\boldsymbol{a}_{\le k})-\frac{1}{2}\boldsymbol{k}^\dagger U_{>k}\Sigma_{>k}U^\dagger_{>k}\boldsymbol{k}-i\boldsymbol{\mu}^\dagger_{>k}U^\dagger_{>k}\boldsymbol{k}} \\
&\propto e^{-\frac{1}{2}\mathbf{a}^\dagger_{\le k}\Sigma^{-1}_{\le k}\mathbf{a}_{\le k}+\boldsymbol{\mu}^\dagger_{\le k}\Sigma^{-1}_{\le k}\mathbf{a}_{\le k}}\int\frac{d\boldsymbol{k}}{(2\pi)^d}e^{-\frac{1}{2}\boldsymbol{k}^\dagger U_{>k}\Sigma_{>k}U^\dagger_{>k}\boldsymbol{k}+i\left(\boldsymbol{y}-U_{\le k}\boldsymbol{a}_{\le k}-U_{>k}\boldsymbol{\mu}_{>k}\right)^\dagger\boldsymbol{k}} \\
&\propto e^{-\frac{1}{2}\mathbf{a}^\dagger_{\le k}\Sigma^{-1}_{\le k}\mathbf{a}_{\le k}+\boldsymbol{\mu}^\dagger_{\le k}\Sigma^{-1}_{\le k}\mathbf{a}_{\le k}+\frac{1}{2}\left(\boldsymbol{y}-U_{\le k}\boldsymbol{a}_{\le k}-U_{>k}\boldsymbol{\mu}_{>k}\right)^\dagger\left(U_{>k}\Sigma_{>k}U^\dagger_{>k}\right)^{-1}\left(\boldsymbol{y}-U_{\le k}\boldsymbol{a}_{\le k}-U_{>k}\boldsymbol{\mu}_{>k}\right)} \\
&\propto e^{-\frac{1}{2}\mathbf{a}^\dagger_{\le k}\Sigma^{-1}_{\le k}\mathbf{a}_{\le k}+\boldsymbol{\mu}^\dagger_{\le k}\Sigma^{-1}_{\le k}\mathbf{a}_{\le k}+\frac{1}{2}\left(\boldsymbol{y}-U_{\le k}\boldsymbol{a}_{\le k}-U_{>k}\boldsymbol{\mu}_{>k}\right)^\dagger U_{>k}\Sigma^{-1}_{>k}U^\dagger_{>k}\left(\boldsymbol{y}-U_{\le k}\boldsymbol{a}_{\le k}-U_{>k}\boldsymbol{\mu}_{>k}\right)}
\end{aligned}
$$

Now it is essential to isolate the terms depending on $\boldsymbol{a}_k$ alone in order to being able to compute the first and second derivatives to define mean and variance of the gaussian. In the following, indices i,j refer to block elements of the matrices they are applied to, while the term *constant* is used for terms that do not explicitely depend on $\mathbf{a}_k$. Let's analyze each term in the exponential of the marginal distribution:

$$
-\frac{1}{2}\mathbf{a}^\dagger_{\le k}\Sigma^{-1}_{\le k}\mathbf{a}_{\le k} = -\frac{1}{2}\sum_{ij}(\boldsymbol{a}_{\le k})_i\left(\Sigma^{-1}_{\le k}\right)_{ij}(\boldsymbol{a}_{\le k})_j = -\frac{1}{2}\boldsymbol{a}^\dagger_k\left(\Sigma^{-1}\right)_k\boldsymbol{a}_k - \sum_{i=1}^{k-1}\boldsymbol{a}^\dagger_i\left(\Sigma^{-1}\right)_{ik}\boldsymbol{a}_{\boldsymbol{k}} + \text{constant}
$$

$$
\boldsymbol{\mu}^\dagger_{\le k}\Sigma^{-1}_{\le k}\mathbf{a}_{\le k} = \sum_{i=1}^{k}\boldsymbol{\mu}^\dagger_i\left(\Sigma^{-1}\right)_{ik}\mathbf{a}_k + \text{constant}
$$

$$\frac{1}{2}\left(\boldsymbol{y}-U_{\leq k}\boldsymbol{a}_{\leq k}-U_{>k}\boldsymbol{\mu}_{>k}\right)^{\dagger}U_{>k}\Sigma_{a,>k}^{-1}U_{>k}^{\dagger}\left(\boldsymbol{y}-U_{\leq k}\boldsymbol{a}_{\leq k}-U_{>k}\boldsymbol{\mu}_{>k}\right)=$$
$$-\boldsymbol{y}^{\dagger}U_{>k}\Sigma_{>k}^{-1}U_{>k}^{\dagger}U_{\leq k}\boldsymbol{a}_{\leq k}+\frac{1}{2}\boldsymbol{a}_{\leq k}^{\dagger}U_{\leq k}^{\dagger}U_{>k}\Sigma_{>k}^{-1}U_{>k}^{\dagger}U_{\leq k}\boldsymbol{a}_{\leq k}+\boldsymbol{a}_{\leq k}^{\dagger}U_{\leq k}^{\dagger}U_{>k}\Sigma_{>k}^{-1}\boldsymbol{\mu}_{>k}+\text{constant}=$$
$$-\sum_{i=1}^{k}\boldsymbol{y}^{\dagger}U_{>k}\Sigma_{>k}^{-1}U_{>k}^{\dagger}U_{i}\boldsymbol{a}_{k}+\frac{1}{2}\boldsymbol{a}_{k}^{\dagger}U_{k}^{\dagger}U_{>k}\Sigma_{a,>k}^{-1}U_{>k}^{\dagger}U_{k}\boldsymbol{a}_{k}+\sum_{i=1}^{k-1}\boldsymbol{a}_{i}^{\dagger}U_{i}^{\dagger}U_{>k}\Sigma_{>k}^{-1}U_{>k}^{\dagger}U_{k}\boldsymbol{a}_{k}+\sum_{i=1}^{k}\boldsymbol{\mu}_{>k}^{\dagger}\Sigma_{>k}^{-1}U_{>k}^{\dagger}U_{i}\boldsymbol{a}_{k}+\text{constant}$$

Computing the first derivative with respect to $\boldsymbol{a}_k$ of the argument of the exponential and setting it equal to zero, we get:

$$\frac{d}{d\boldsymbol{a}_k}\Big[-\frac{1}{2}\mathbf{a}_{\leq k}^{\dagger}\Sigma_{\leq k}^{-1}\mathbf{a}_{\leq k}+\boldsymbol{\mu}_{\leq k}^{\dagger}\Sigma_{\leq k}^{-1}\mathbf{a}_{\leq k}+$$
$$+\frac{1}{2}\left(\boldsymbol{y}-U_{\leq k}\boldsymbol{a}_{\leq k}-U_{>k}\boldsymbol{\mu}_{>k}\right)^{\dagger}U_{>k}\Sigma_{>k}^{-1}U_{>k}^{\dagger}\left(\boldsymbol{y}-U_{\leq k}\boldsymbol{a}_{\leq k}-U_{>k}\boldsymbol{\mu}_{>k}\right)\Big]=$$
$$=-\left(\Sigma^{-1}\right)_{k}\boldsymbol{a}_{k}-\sum_{i=1}^{k}\left(\boldsymbol{y}^{\dagger}U_{>k}\Sigma_{>k}^{-1}U_{>k}^{\dagger}U_{i}\right)^{\dagger}+$$
$$+\sum_{i=1}^{k-1}\left[\left(\boldsymbol{a}_{i}^{\dagger}U_{i}^{\dagger}U_{>k}\Sigma_{>k}^{-1}U_{>k}^{\dagger}U_{k}\right)^{\dagger}+\left(\boldsymbol{\mu}_{>k}^{\dagger}\Sigma_{>k}^{-1}U_{>k}^{\dagger}U_{i}\right)^{\dagger}-\sum_{i=1}^{k-1}\left(\boldsymbol{a}_{i}^{\dagger}\left(\Sigma^{-1}\right)_{ik}\right)^{\dagger}\right]+U_{k}^{\dagger}U_{>k}\Sigma_{>k}^{-1}U_{>k}^{\dagger}U_{k}\boldsymbol{a}_{k}$$
$$-\left(\Sigma^{-1}\right)_{k}\boldsymbol{a}_{k}-\sum_{i=1}^{k}U_{i}^{\dagger}U_{>k}\Sigma_{>k}^{-1}U_{>k}^{\dagger}\boldsymbol{y}+$$
$$+\sum_{i=1}^{k-1}\left[U_{k}^{\dagger}U_{>k}\Sigma_{>k}^{-1}U_{>k}^{\dagger}U_{i}\boldsymbol{a}_{i}+U_{i}^{\dagger}U_{>k}\Sigma_{>k}^{-1}\boldsymbol{\mu}_{>k}-\left(\Sigma^{-1}\right)_{ik}\boldsymbol{a}_{i}\right]+U_{k}^{\dagger}U_{>k}\Sigma_{>k}^{-1}U_{>k}^{\dagger}U_{k}\boldsymbol{a}_{k}=0$$

which gives a mean $\boldsymbol{\mu}_{\boldsymbol{a}_k|\boldsymbol{a}_{<k},\boldsymbol{y}}$ given by:

$$\boldsymbol{\mu}_{\boldsymbol{a}_k|\boldsymbol{a}_{<k},\boldsymbol{y}}:=\boldsymbol{a}_k^{*}=$$
$$=\left[\left(\Sigma^{-1}\right)_{k}-U_{k}^{\dagger}U_{>k}\Sigma_{>k}^{-1}U_{>k}^{\dagger}U_{k}\right]^{-1}\left\{\sum_{i=1}^{k-1}\left[U_{k}^{\dagger}U_{>k}\Sigma_{>k}^{-1}U_{>k}^{\dagger}U_{i}\boldsymbol{a}_{i}+U_{i}^{\dagger}U_{>k}\Sigma_{>k}^{-1}\boldsymbol{\mu}_{>k}-\left(\Sigma^{-1}\right)_{ik}\boldsymbol{a}_{i}\right]-\sum_{i=1}^{k}U_{i}^{\dagger}U_{>k}\Sigma_{>k}^{-1}U_{>k}^{\dagger}\boldsymbol{y}\right\}$$
$$=\Sigma_{\boldsymbol{a}_k|\boldsymbol{a}_{<k},\boldsymbol{y}}\left\{\sum_{i=1}^{k-1}\left[U_{k}^{\dagger}U_{>k}\Sigma_{>k}^{-1}U_{>k}^{\dagger}U_{i}\boldsymbol{a}_{i}+U_{i}^{\dagger}U_{>k}\Sigma_{>k}^{-1}\boldsymbol{\mu}_{>k}-\left(\Sigma^{-1}\right)_{ik}\boldsymbol{a}_{i}\right]-\sum_{i=1}^{k}U_{i}^{\dagger}U_{>k}\Sigma_{>k}^{-1}U_{>k}^{\dagger}\boldsymbol{y}\right\}$$

The second derivative returns the inverse of the covariance matrix

$$\Sigma_{\boldsymbol{a}_k|\boldsymbol{a}_{<k},\boldsymbol{y}}^{-1}:=-\frac{d^2}{d\boldsymbol{a}_k^2}\left[-\frac{1}{2}\mathbf{a}_{\leq k}^{\dagger}\Sigma_{\leq k}^{-1}\mathbf{a}_{\leq k}+\boldsymbol{\mu}_{\leq k}^{\dagger}\Sigma_{\leq k}^{-1}\mathbf{a}_{\leq k}+\right.$$
$$\left.+\frac{1}{2}\left(\boldsymbol{y}-U_{\leq k}\boldsymbol{a}_{\leq k}-U_{>k}\boldsymbol{\mu}_{>k}\right)^{\dagger}U_{>k}\Sigma_{>k}^{-1}U_{>k}^{\dagger}\left(\boldsymbol{y}-U_{\leq k}\boldsymbol{a}_{\leq k}-U_{>k}\boldsymbol{\mu}_{>k}\right)\right]=$$
$$=-\frac{d}{d\boldsymbol{a}_k}\Big[-\left(\Sigma^{-1}\right)_{k}\boldsymbol{a}_{k}-\sum_{i=1}^{k}U_{i}^{\dagger}U_{>k}\Sigma_{>k}^{-1}U_{>k}\boldsymbol{y}+$$
$$+\sum_{i=1}^{k-1}\left[U_{k}^{\dagger}U_{>k}\Sigma_{>k}^{-1}U_{>k}^{\dagger}U_{i}\boldsymbol{a}_{i}+U_{i}^{\dagger}U_{>k}\Sigma_{>k}^{-1}\boldsymbol{\mu}_{>k}-\left(\Sigma^{-1}\right)_{ik}\boldsymbol{a}_{i}\right]+U_{k}^{\dagger}U_{>k}\Sigma_{>k}^{-1}U_{>k}^{\dagger}U_{k}\boldsymbol{a}_{k}\Big]$$
$$=\left(\Sigma^{-1}\right)_{k}-U_{k}^{\dagger}U_{>k}\Sigma_{>k}^{-1}U_{>k}^{\dagger}U_{k}$$

Having characterized the k-th marginal, we have a solid foundation to define the k-th term in the autoregressive

decomposition of the full joint distribution $P(\mathbf{a}, \mathbf{y})$:

$$
\begin{aligned}
P(\boldsymbol{a}_k|\boldsymbol{a}_{<k}, y) =& \frac{1}{\sqrt{(2\pi)^q \det(\Sigma_{\boldsymbol{a}_k|\boldsymbol{a}_{<k},\boldsymbol{y}})}} e^{-\frac{1}{2}(\boldsymbol{a}_k - \boldsymbol{\mu}_{\boldsymbol{a}_k|\boldsymbol{a}_{<k},\boldsymbol{y}})^\dagger \Sigma^{-1}_{\boldsymbol{a}_k|\boldsymbol{a}_{<k},\boldsymbol{y}} (\boldsymbol{a}_k - \boldsymbol{\mu}_{\boldsymbol{a}_k|\boldsymbol{a}_{<k},\boldsymbol{y}})} \\
=& \frac{e^{\mu^\dagger_{\boldsymbol{a}_k|\boldsymbol{a}_{<k},\boldsymbol{y}} \Sigma^{-1}_{\boldsymbol{a}_k|\boldsymbol{a}_{<k},\boldsymbol{y}} \mu_{\boldsymbol{a}_k|\boldsymbol{a}_{<k},\boldsymbol{y}}}}{\sqrt{(2\pi)^q \det(\Sigma_{\boldsymbol{a}_k|\boldsymbol{a}_{<k},\boldsymbol{y}})}} e^{-\frac{1}{2}\boldsymbol{a}_k^\dagger \Sigma^{-1}_{\boldsymbol{a}_k|\boldsymbol{a}_{<k},\boldsymbol{y}} \boldsymbol{a}_k + \boldsymbol{a}_k^\dagger \Sigma^{-1}_{\boldsymbol{a}_k|\boldsymbol{a}_{<k},\boldsymbol{y}} \boldsymbol{\mu}_{\boldsymbol{a}_k|\boldsymbol{a}_{<k},\boldsymbol{y}}} \\
\propto& e^{-\frac{1}{2}\boldsymbol{a}_k^\dagger \Sigma^{-1}_{\boldsymbol{a}_k|\boldsymbol{a}_{<k},\boldsymbol{y}} \boldsymbol{a}_k + \boldsymbol{a}_k^\dagger \left\{\sum_{i=1}^{k-1}\left[U_k^\dagger U_{>k} \Sigma^{-1}_{>k} U^\dagger_{>k} U_i \boldsymbol{a}_i + U_i^\dagger U_{>k} \Sigma^{-1}_{>k} \boldsymbol{\mu}_{>k} - \left(\Sigma^{-1}\right)_{ik} \boldsymbol{a}_i\right] - \sum_{i=1}^{k} U_i^\dagger U_{>k} \Sigma^{-1}_{a,>k} U^\dagger_{>k} \boldsymbol{y}\right\}} \\
=& \frac{1}{Z_{\boldsymbol{a}_k|\boldsymbol{a}_{<k},\boldsymbol{y}}} e^{-H(\boldsymbol{a}_k|\boldsymbol{a}_{<k},\boldsymbol{y})}
\end{aligned}
$$

where the energy function $H(\mathbf{a}_k|\mathbf{a}_{<k}, \mathbf{y})$ can be written as:

$$
\begin{aligned}
&H(\boldsymbol{a}_k|\boldsymbol{a}_{<k}, y) = \frac{1}{2}\boldsymbol{a}_k^\dagger \Sigma^{-1}_{\boldsymbol{a}_k|\boldsymbol{a}_{<k},\boldsymbol{y}} \boldsymbol{a}_k + \\
&-\boldsymbol{a}_k^\dagger \left\{ \sum_{i=1}^{k-1} \left[U_k^\dagger U_{>k} \Sigma^{-1}_{>k} U^\dagger_{>k} U_i \boldsymbol{a}_i + U_i^\dagger U_{>k} \Sigma^{-1}_{>k} \boldsymbol{\mu}_{>k} - \left(\Sigma^{-1}\right)_{ik} \boldsymbol{a}_i \right] - \sum_{i=1}^{k} U_i^\dagger U_{>k} \Sigma^{-1}_{a,>k} U^\dagger_{>k} \boldsymbol{y} \right\} = \\
&= \frac{1}{2}\boldsymbol{a}_k^\dagger \Sigma^{-1}_{\boldsymbol{a}_k|\boldsymbol{a}_{<k},\boldsymbol{y}} \boldsymbol{a}_k + \boldsymbol{a}_k^\dagger \sum_{i=1}^{k-1} \left[\left(\Sigma^{-1}\right)_{ik} - U_k^\dagger U_{>k} \Sigma^{-1}_{>k} U^\dagger_{>k} U_i \right] \boldsymbol{a}_i - \boldsymbol{a}_k^\dagger \sum_{i=1}^{k-1} U_i^\dagger U_{>k} \Sigma^{-1}_{>k} \boldsymbol{\mu}_{>k} + \boldsymbol{a}_k^\dagger \sum_{i=1}^{k} U_i^\dagger U_{>k} \Sigma^{-1}_{a,>k} U^\dagger_{>k} \boldsymbol{y}
\end{aligned}
$$

Discrete model

Now that we have a principled formulation of the energy function of the $\boldsymbol{k}-$th term in the autoregressive decomposition, we can restore the discrete nature of the amino-acid data and produce an ansatz for a discrete version of the model in its autoregressive implementation. Let's consider each of the three contributions in the energy function:

1) Quadratic term in the amino-acid space

$$
\frac{1}{2}\boldsymbol{a}_k^\dagger \Sigma^{-1}_{\boldsymbol{a}_k|\boldsymbol{a}_{<k},\boldsymbol{y}} \boldsymbol{a}_k + \boldsymbol{a}_k^\dagger \sum_{i=1}^{k-1} \left[\left(\Sigma^{-1}\right)_{ik} - U_k^\dagger U_{>k} \Sigma^{-1}_{>k} U^\dagger_{>k} U_i \right] \boldsymbol{a}_i
$$

This can be thought of as an interaction term:

$$
\boldsymbol{a}^\dagger_{\leq k} J_{\leq k} \boldsymbol{a}_{\leq k} \qquad \begin{cases} \boldsymbol{a}_{\leq k} \in [0,1]^{qk} & \text{is the one-hot sub-vector representing the first } k \text{ positions of the sequence} \\ J_{\leq k} \in \mathbb{R}^{qk,qk} & \text{is an interaction matrix composed of } q \times q \text{ blocks} \end{cases}
$$

Using a *one-cold* representation where $\boldsymbol{a} \in \{0, 1, 2, \ldots, q\}^L$, the same can be written as

$$
\sum_{k<i} J_{ik}(a_i, a_k)
$$

2) Linear term in the the amino-acid space

$$
\boldsymbol{a}_k^\dagger \sum_{i=1}^{k-1} U_i^\dagger U_{>k} \Sigma^{-1}_{>k} \boldsymbol{\mu}_{>k}
$$

This can be easily interpreted as a local field. In a one-hot representation this is $\boldsymbol{a}_k^\dagger \boldsymbol{h}_k$ with $\boldsymbol{h}_k \in \mathbb{R}^q$, while in a *one-cold* representation this is

$$
h_k(a_k).
$$

3) Cross interaction term between the amino-acid space and the principal component space:

$$
\boldsymbol{a}_k^\dagger \sum_{i=1}^{k} U_i^\dagger U_{>k} \Sigma^{-1}_{a,>k} U^\dagger_{>k} \boldsymbol{y}
$$

This can be written as

$$\boldsymbol{a}_k^\dagger G \boldsymbol{y} \qquad G \in \mathbb{R}^{q,d}$$

where G acts as an embedding mapping from the amino-acid space to the principal component space. In a *one-cold* representation, this is

$$G(a_k) \cdot \boldsymbol{y}$$

The full one-cold energy function thus reads:

$$H(a_k|a_{<k}, \boldsymbol{y}) = -\sum_{i<k} J_{ki}(a_k, a_i) - h_k(a_k) - \mathbf{y} \cdot \boldsymbol{G}_k(a_k)$$

and the probability distribution of the k-th term is

$$\begin{aligned} P(a_k|a_{<k}, \boldsymbol{y}) &= \frac{1}{Z_{a_k|a_{<k},\boldsymbol{y}}} e^{\sum_{i<k} J_{ki}(a_k,a_i)+h_k(a_k)+\mathbf{y}\cdot\boldsymbol{G}_k(a_k)} \\ &= \frac{e^{\sum_{i<k} J_{ki}(a_k,a_i)+h_k(a_k)+\mathbf{y}\cdot\boldsymbol{G}_k(a_k)}}{\sum_{c=1}^{q} e^{\sum_{i<k} J_{ki}(c,a_i)+h_k(c)+\mathbf{y}\cdot\boldsymbol{G}_k(c)}} \end{aligned}$$

$$Z_{a_k|a_{<k},\boldsymbol{y}} = \sum_{c=1}^{q} e^{\sum_{i<k} J_{ki}(c,a_i)+h_k(c)+\mathbf{y}\cdot\boldsymbol{G}_k(c)}$$

Given a MSA $\mathcal{D} \in \{1, \ldots, q\}^{L,M} = \{a_i^m\}$ concatenated to a matrix $\boldsymbol{Y} \in \mathbb{R}^{d,M} = \{y_\alpha^m\}$ representing the principal components of each sequence in $\mathcal{D}$, the likelihood of the model can be written as

$$\mathcal{L} = \frac{1}{M}\sum_{m=1}^{M} \log P(\boldsymbol{a}^m, \boldsymbol{y}^m) = \frac{1}{M}\sum_{m=1}^{M} \left\{ \log P(\boldsymbol{y}^m) + \log P(a_1^m|\boldsymbol{y}^m) + \sum_{k=2}^{L} \log P(a_k^m|\boldsymbol{a}_{<k}^m, \boldsymbol{y}^m) \right\}$$

The first term of the likelihood can be thought of as an empirical frequency of the sequences in the PC space. Since it does not depend on the learning parameters of the model, it can be discarded.

$$\begin{aligned} \mathcal{L} = \frac{1}{M}\sum_{m=1}^{M} \Bigg\{ & h_1(a_1^m) + G_1(a_1^m) \cdot \boldsymbol{y}^m + \log \sum_{c=1}^{q} e^{h_i(c)+\mathbf{y}^m\cdot G_1(c)} + \\ & + \sum_{k=2}^{L} \left[\sum_{i<k} J_{ki}(a_k^m, a_i^m) + h_k(a_k^m) + \mathbf{y}^m \cdot G_k(a_k^m) - \log \sum_{c=1}^{q} e^{\sum_{i<k} J_{ki}(c,a_i^m)+h_k(c)+\mathbf{y}^m\cdot G_k(c)} \right] \Bigg\} = \\ = \frac{1}{M}\sum_{k=1}^{L}\sum_{m=1}^{M} & \mathcal{L}_k^m(\boldsymbol{a}^m, \boldsymbol{y}^m | \{\boldsymbol{h}_k, J_{k,*}, G_k\}) \end{aligned}$$

Since the parameters relative to each site of the sequence are factorized, the full likelihood can be optimized in parallel. So, each single optimization problem is defined by the following objective function:

$$\mathcal{L}_k(\mathcal{D}|\{\boldsymbol{h}_k, J_{k,*}, G_k\}) = \\ = \begin{cases} \frac{1}{M}\sum_{m=1}^{M} \left\{ h_1(a_1^m) + G_1(a_1^m) \cdot \boldsymbol{y}^m - \log \sum_{c=1}^{q} e^{h_i(c)+\mathbf{y}^m\cdot G_1(c)} \right\} & k = 1 \\ \frac{1}{M}\sum_{m=1}^{M} \left\{ \sum_{i<k} J_{ki}(a_k^m, a_i^m) + h_k(a_k^m) + \mathbf{y}^m \cdot \boldsymbol{G}_k(a_k^m) - \log \sum_{c=1}^{q} e^{\sum_{i<k} J_{ki}(c,a_i^m)+h_k(c)+\mathbf{y}^m\cdot G_k(c)} \right\} & k \geq 2 \end{cases}$$

APPENDIX A3: IMPLEMENTATION DETAILS

The model architecture and all computational experiments were implemented in the Julia programming language. The full source code, along with documentation and scripts to reproduce the results, is available in a public GitHub repository `https://github.com/francescocaredda/FeatureDCA.jl`.

Model training proceeds by minimizing the negative log-likelihood of the conditional distribution at each position k, corresponding to the autoregressive factorization described in Eq. (1) of the main text. The objective function was optimized using deterministic gradient descent with the `LD_LBFGS` algorithm [3] provided by the `NLopt.jl` package [4]. Optimization was performed with a convergence tolerance of 10^{-5}. We applied L_2 regularization to three model components: the local fields $h_i(a)$, the interaction tensors $J_{ij}(a,b)$, and the feature embedding matrix G. A single regularization coefficient of $\lambda = 10^{-4}$ was used across all terms.

Principal component analysis (PCA) was conducted using the `MultivariateStats.jl` package. Multiple sequence alignments (MSAs) were one-hot encoded and centered prior to PCA. The top d principal components were retained and used as conditioning inputs throughout training and generation.

Training and sampling can be parallelized across sequences or positional blocks due to the factorized structure of the model. Sinkhorn divergences were computed using a custom Julia implementation of the Sinkhorn–Knopp algorithm [5] in the `OptimalTransport.jl` package.

APPENDIX A4: DATA PROCESSING AND MSA CONSTRUCTION

The analyses presented in the *Generativity* section of the main text were carried out using the multiple sequence alignments (MSAs) available at `https://github.com/pagnani/ArDCAData`, which were originally curated for the benchmarking of ArDCA [6].

For the analysis of the Response Regulator (RR) family (Pfam ID PF00072), we constructed a custom MSA to capture the structural diversity across its known functional subclasses. We first retrieved from UniProt all sequences exhibiting a two-domain architecture composed of the conserved receiver domain PF00072 in combination with one of the three DNA-binding domains that define the major RR subclasses: PF00486 (Trans_Reg_C), PF04397 (LytTR), and PF00196 (GerE). This yielded a total of 160,585 non-redundant sequences. To ensure structural relevance and compatibility with available experimental structures, we used the `jackhmmer` tool to identify the 200 sequences in UniProt most similar to the RR representatives in the Protein Data Bank: 1NXS (Trans_Reg_C), 4CBV (LytTR), and 4ZMS (GerE). These sequences were used to construct a profile Hidden Markov Model (HMM) using the `hmmbuild` tool from the HMMER suite [7]. The resulting HMM spanned 118 aligned positions and was used as a reference for alignment. All 160,585 RR sequences were then aligned to the profile HMM using `hmmalign`. Insertions relative to the HMM model were removed to ensure consistency in positional indexing, resulting in a clean, fixed-length MSA. This alignment was used to train FeatureDCA for the generation of class-specific RR sequences as described in the section *The case of RR homodimers* of the main text. Data relative to the study of RR homodimers is available at `https://github.com/francescocaredda/FeatureDCAData`

TABLE A1. Summary of the protein families used throughout the analysis. Reported are the Pfam identifier, a brief description, the sequence length L, the number of sequences M in the multiple sequence alignment, and the number of parameters to train the model in the limits of $d = 2$ and $d = 512$.

Name	Description	L	M	#parameters
PF00014	Kunitz Domain	53	8,871	0.6-1.1M
PF00072	Response Regulator (RR)	112	574,565	2.7-3.9M
PF00072	RR, Homodimers Analysis	118	264,684	3.0-4.3M
PF00076	RRM	70	79,366	1.0-1.8M
PF00595	PDZ Domain	82	15,299	1.4-2.3M
PF13354	Beta-Lactamase	202	7,515	8.9-1.1M
PF13354	Beta-Lactamase, DMS Analysis	214	58,021	10-12M

APPENDIX A5: WASSERSTEIN DISTANCE AND SINKHORN DIVERGENCE

Comparing the PCA projections of natural and generated sequences is a common step in evaluating generative models for aligned protein families. This comparison is often performed qualitatively, by inspecting two-dimensional projections and assessing whether the generated sequences reproduce the overall geometry, clustering, and density of the natural distribution. To complement this visual inspection with a rigorous, quantitative metric, we use the Wasserstein distance, a concept originating from Optimal Transport theory, which provides a means to measure the distance between two probability distributions over a geometric space.

Consider two distributions of points $\mathbf{X} = \{\mathbf{x}_1, \ldots, \mathbf{x}_M\}$ and $\mathbf{Y} = \{\mathbf{y}_1, \ldots, \mathbf{y}_M\}$, living in the same Euclidean space

$\mathbb{R}^N$, equipped with the standard Euclidean metric:

$$d(\mathbf{x}, \mathbf{y}) = \sqrt{\sum_{i=1}^{N} (x_i - y_i)^2}.$$

We construct a cost matrix $\mathbf{C} \in \mathbb{R}^{M \times M}$, where each entry $C_{ij} = d(\mathbf{x}_i, \mathbf{y}_j)^2$ represents the squared cost of transporting unit mass from $\mathbf{x}_i \in \mathbf{X}$ to $\mathbf{y}_j \in \mathbf{Y}$. Let μ and ν be uniform discrete probability measures over $\mathbf{X}$ and $\mathbf{Y}$, respectively, defined as

$$\mu = \frac{1}{M} \sum_{i=1}^{M} \delta_{\mathbf{x}_i}$$

$$\nu = \frac{1}{M} \sum_{j=1}^{M} \delta_{\mathbf{y}_j},$$

where $\delta_{\mathbf{x}}$ denotes the Dirac measure centered at $\mathbf{x}$.

The Wasserstein distance of order 2 (also called the Earth Mover's Distance) is defined as the minimum total cost of transporting μ to ν, subject to mass conservation:

$$W_2^2(\mu, \nu) = \min_{\gamma \in \Gamma(\mu,\nu)} \sum_{i=1}^{M} \sum_{j=1}^{M} \gamma_{ij} C_{ij},$$

where $\Gamma(\mu, \nu)$ is the set of joint probability distributions (transport plans) with marginals μ and ν. In this setting, γ represents the optimal transport plan and $\gamma_{ij} C_{ij}$ is the amount of probability mass transported from $\mathbf{x}_i$ to $\mathbf{y}_j$.

The Wasserstein distance provides a natural and geometrically grounded way to compare distributions over vector spaces. However, computing it exactly for empirical datasets can be computationally expensive and numerically unstable, particularly as the number of samples grows. These drawbacks are effectively solved by the concept of Sinkhorn divergence, a regularized version of the Wasserstein distance that preserves its core geometric meaning.

Given two discrete distributions μ and ν supported on point clouds $\mathbf{X}$ and $\mathbf{Y}$, and a cost matrix $\mathbf{C}$ as defined above, the entropically regularized transport cost is given by:

$$\mathrm{OT}_\varepsilon(\mu, \nu) = \min_{\gamma \in \Gamma(\mu,\nu)} \sum_{i,j} \gamma_{ij} C_{ij} + \varepsilon KL(\gamma || \mu \otimes \nu),$$

where $KL(\gamma || \mu \otimes \nu) = \sum_{i,j} \gamma_{ij} \log \frac{\gamma_{ij}}{\mu_i \nu_j}$ is the Kullback-Leibler penalty that favours factorized optimal plans γ, and $\varepsilon > 0$ is a regularization parameter controlling the trade-off between accuracy and smoothness. However, this regularized transport cost is not a proper metric: it does not satisfy symmetry or the triangle inequality, and it may not vanish when $\mu = \nu$. The Sinkhorn divergence resolves this by symmetrizing and correcting the regularized cost:

$$S_\varepsilon(\mu, \nu) = \mathrm{OT}_\varepsilon(\mu, \nu) - \frac{1}{2} \left[\mathrm{OT}_\varepsilon(\mu, \mu) + \mathrm{OT}_\varepsilon(\nu, \nu) \right].$$

This expression ensures that $S_\varepsilon(\mu, \nu) = 0$ if and only if $\mu = \nu$, and retains the geometry of the Wasserstein space while benefiting from better numerical stability and computational efficiency. The Sinkhorn divergence can be efficiently computed using iterative matrix scaling algorithms (e.g., Sinkhorn–Knopp) and is particularly well-suited to comparing empirical distributions like the PCA projections of natural and generated sequences [5].

In our analysis, we use the Sinkhorn divergence with squared Euclidean cost and uniform weights over the empirical samples, as implemented in the Julia `OptimalTransport.jl` library, setting the regularization parameter $\epsilon = 0.05$.

APPENDIX A6: PRINCIPAL COMPONENTS HIGHER THAN THE SECOND

Traditional autoregressive and energy-based models such as ArDCA and bmDCA are trained without any explicit access to global, low-dimensional features of the sequence distribution. While they often reproduce the marginal

statistics and low-rank structure of the MSA, they cannot selectively capture higher-order variation, especially along principal components beyond the first few. In practice, this means that although these models can approximate the dominant modes of variation, typically PC1 and PC2, they fail to reproduce finer, structured diversity in the MSA, which is often functionally or structurally meaningful.

In contrast, FeatureDCA incorporates a tunable number of principal components as conditioning inputs, allowing the model to explicitly learn and generate along increasingly complex directions of evolutionary variation. This flexibility enables FeatureDCA to match the natural sequence distribution not just along the first few PCs, but also across mid- and higher-rank components, depending on the dimensionality d used during training.

Fig A1 illustrates this effect for family PF13354. It compares the projections of natural and generated sequences across the first ten principal components (shown pairwise as PC1 vs PC2, PC3 vs PC4, ..., PC9 vs PC10). Sequences generated by ArDCA and bmDCA align well with the natural data only in the first projection, while diverging noticeably in higher PC pairs. In contrast, FeatureDCA, when trained with increasing numbers of PCs ($d = 2, 4, 8, 16$), shows progressively improved agreement across the full range of components, demonstrating its ability to capture structured variability at different scales.

APPENDIX A7: ADDITIONAL STRUCTURAL ANALYSIS OF THE PF00014 MISMATCH CASE

In order to benchmark the generative capabilities of FeatureDCA at the structural level, it is essential that the structure prediction methods used for evaluation are themselves able to distinguish between distinct folds within the same protein family. To illustrate a case in which this assumption may break down, in Fig 4 of the main text we highlighted the case of the PF00014 family, for which the RMSD between two experimentally determined structures (PDB IDs 1UUB and 1LD6) is substantially larger than the RMSD observed between the ESMFold-predicted structures of the same sequences. This apparent inconsistency raises the question of whether the discrepancy originates from limitations of RMSD as a structural metric, or instead reflects a genuine inability of AF/ESMFold to reproduce experimentally observed fold differences within this family. In this section, we address this point by complementing the RMSD-based comparison with additional topology-aware analyses, aimed at clarifying the origin of the PF00014 mismatch.

PF00014 corresponds to the bovine pancreatic trypsin inhibitor (BPTI) family. The experimental structure 1UUB is an X-ray structure of the wild-type protein determined under standard conditions and serves as a reference for the canonical fold of the family. The structure 1LD6 corresponds to a heavily mutated BPTI variant, obtained by introducing eight alanine substitutions relative to the wild-type structure 4PTI. The structure of 1LD6 was determined by NMR under standard solution conditions and represents an experimentally validated alternative conformation within the same protein family. To further contextualize this mutant, we also considered the structure 1LD5, which corresponds to a closely related variant carrying a single mutation relative to 4PTI. Importantly, the sequences of 4PTI, 1LD5, and 1LD6 have identical length and residue indexing, enabling direct structural comparisons without alignment ambiguities.

For each sequence, we generated predicted structures using the same AF/ESMFold folding pipeline employed throughout the manuscript. We then compared experimental and predicted structures using three complementary metrics: RMSD, TM-score, and contact overlap. In addition to RMSD, which is reported in the main text, we computed the TM-score as a length-normalized measure of global fold similarity that emphasizes overall topology and is less sensitive to local deviations. We also performed a contact-based analysis, defining a contact as a pair of residues whose C_α atoms are separated by less than 8 Å and whose sequence separation exceeds four residues (i.e., $|i-j| > 4$). For each pair of structures, we computed the number of shared contacts, taking advantage of the identical sequence length and indexing across all structures.

Focusing on the 1LD6 sequence, we find that the AF/ESMFold-predicted structure does not preferentially match the experimental 1LD6 conformation. While the RMSD between the predicted structure and experimental 1LD6 is approximately 1.5 Å, the same predicted structure is significantly closer to both 1LD5 and the wild-type 4PTI, with RMSDs of approximately 0.8 Å and 0.2 Å, respectively. This trend is further supported by TM-score analysis: the TM-score between the predicted structure and experimental 1LD6 is approximately 0.69, whereas higher TM-scores are obtained when comparing the predicted structure to 1LD5 (TM-score ≈ 0.85) and to 4PTI (TM-score ≈ 0.98). The contact-based analysis yields a consistent picture, with the predicted structure sharing fewer contacts with experimental 1LD6 (233 shared contacts) than with 1LD5 (252 shared contacts) or 4PTI (263 shared contacts).

Taken together, these results indicate that the discrepancy highlighted in Fig 4 is not an artifact arising from the nonlinear behavior of RMSD. Instead, they show that for the heavily mutated 1LD6 sequence, AF/ESMFold predicts a structure that is topologically closer to the canonical wild-type fold and to minimally mutated variants than to the experimentally determined 1LD6 conformation. More generally, this analysis supports the interpretation presented in the main text: for certain protein families and engineered sequences, current structure prediction methods may

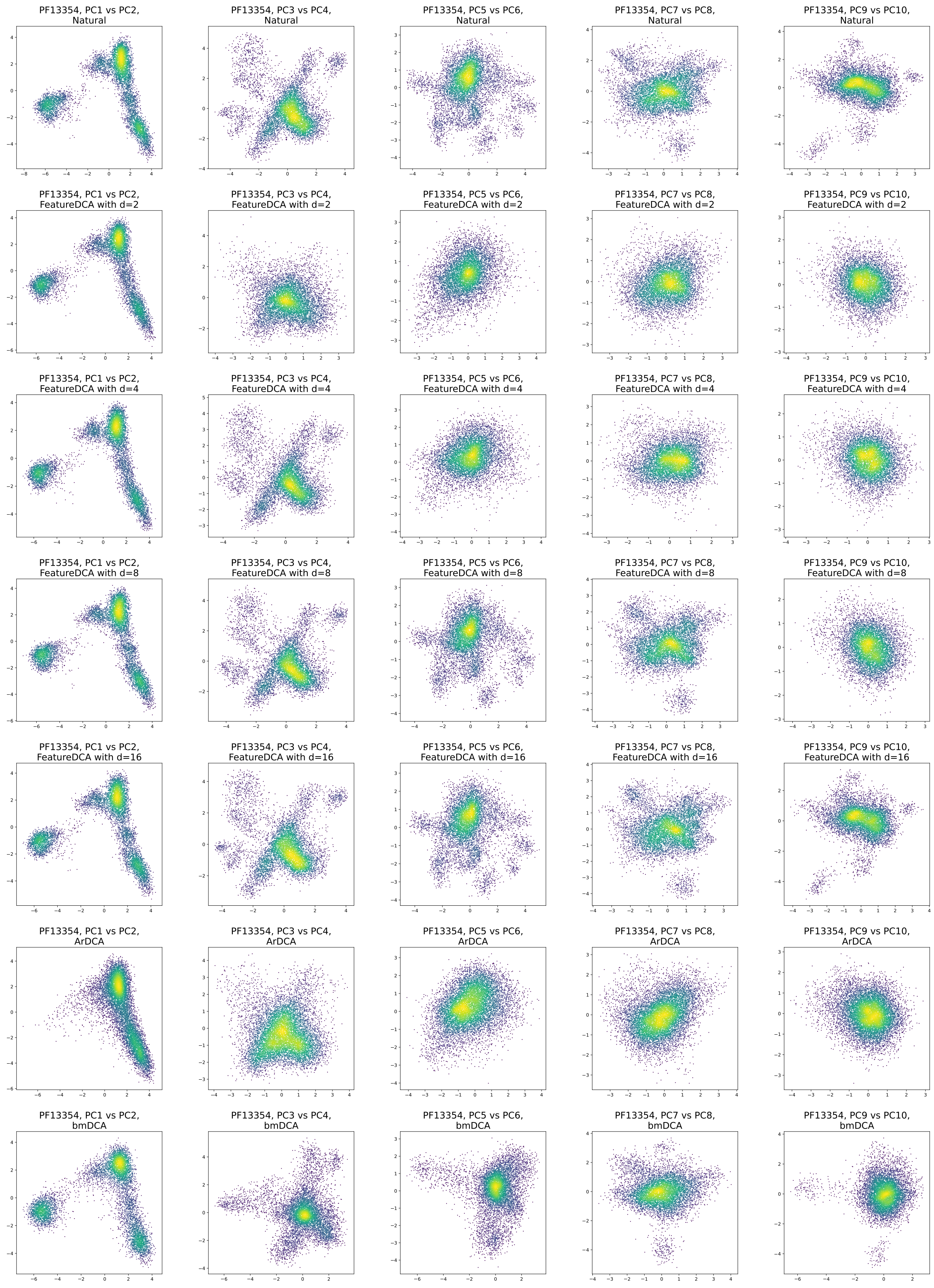

FIG. A1. **Principal component projections of natural and generated sequences for PF13354**. Each column corresponds to a projection onto a pair of principal components: PC1 vs PC2, PC3 vs PC4, ..., up to PC9 vs PC10. The first row shows natural MSA sequences. Subsequent rows display sequences generated by different models: FeatureDCA trained with $d = 2, 4, 8, 16$ principal components, ArDCA, and bmDCA. The figure illustrates that FeatureDCA trained with d PCs is able to faithfully reproduce the natural sequence distribution within the corresponding first d principal components, with progressively improved agreement as d increases.

fail to reproduce experimentally observed fold variations, limiting their use as a benchmark for assessing fold-specific generative modeling such as FeatureDCA.

APPENDIX A8: PLDDT ANALYSIS FOR GENERATED RR HOMODIMER SEQUENCES

To complement the RMSD-based structural evaluation reported in the main text, we analyzed the predicted Local Distance Difference Test (pLDDT) scores associated with AlphaFold predictions of generated sequences for the RR homodimer family. This analysis focuses on the three response regulator classes considered in Section *The case of RR homodimers* and in Figs 5,6, and 7 of the main text, namely Trans_Reg_C, GerE, and LytTR.

For each generated sequence, AlphaFold provides a per-residue pLDDT score, which reflects the internal confidence of the structure prediction model. Here, pLDDT is used solely as an indicator of the overall confidence of the predicted structures and not as a measure of their accuracy with respect to experimental conformations. For each sequence, we computed the mean pLDDT by averaging over all residues. For each protein family and conditioning dimension d, these values were then averaged over the ensemble of generated sequences, and the corresponding standard deviation was recorded.

Fig A2 reports the mean pLDDT as a function of the number of principal components used for conditioning. Across all three RR homodimer classes, the predicted structures are associated with moderate to high pLDDT values over the full range of conditioning dimensions. Notably, the Trans_Reg_C family consistently exhibits the highest mean pLDDT values, typically exceeding 85 and increasing further for larger conditioning dimensions. The GerE family displays intermediate behavior, with mean pLDDT values increasing steadily as the number of principal components grows. In contrast, the LytTR family shows systematically lower mean pLDDT values, although still within a range indicative of reasonably confident predictions.

This hierarchy among the three RR homodimer classes mirrors the trends observed in the RMSD-based structural evaluation in the main text, where Trans_Reg_C displays the most consistent folding behavior and LytTR represents the most challenging case. Overall, this analysis shows that the RMSD trends reported for RR homodimers do not arise from low-confidence AlphaFold predictions, but instead reflect consistent structural differences in confidently predicted folds.

APPENDIX A9: IN-SILICO DEEP MUTATIONAL SCANNING

To evaluate the ability of FeatureDCA to predict mutational effects, we performed an in-silico deep mutational scanning (DMS) experiment. The predictive score for a mutation at position i, from amino acid a to amino acid b, is computed as the difference in log-likelihood between the mutant and the wild-type sequence:

$$\Delta E(a_i \to b_i) = -\log P(\boldsymbol{a}^{wt}, \boldsymbol{y}) + \log P(\boldsymbol{a}^{mut}, \tilde{\boldsymbol{y}}) = -\log \frac{P(a_1, ..., a_{i-1}, b_i, a_{i+1}, ..., a_L, \tilde{\boldsymbol{y}})}{P(a_1, ..., a_{i-1}, a_i, a_{i+1}, ..., a_L, \boldsymbol{y})},$$

where $\boldsymbol{a}^{wt}$ is the wild-type sequence and $\boldsymbol{a}^{mut}$ is the same sequence with residue a at position i replaced by residue b, while $\boldsymbol{y}$ and $\tilde{\boldsymbol{y}}$ are the PC projections of the wild-type and mutant sequence, respectively. The probabilities $P(\boldsymbol{a}, \boldsymbol{y})$ are obtained directly from the model's autoregressive probability decomposition.

The experimental DMS measurements used for comparison were taken from the Excel file available from `https://static-content.springer.com/esm/art%3A10.1038%2Fs41592-018-0138-4/MediaObjects/41592_2018_138_MOESM4_ESM.xlsx` [8]. We used the data corresponding to the `blat_ecoli` dataset, which refers to the TEM-1 beta-lactamase experiment by Ostermeier and collaborators [9]. To ensure consistency between model predictions and experimental values, we preprocessed the Ostermeier dataset as follows. First, we alphabetically ordered the amino acid substitutions at each site (A, C, D, E, ..., Y) for reproducibility. For any missing single-point mutation, we explicitly added an entry with a placeholder mutational score of `inf`, representing absence of data. We then constructed a multiple sequence alignment (MSA) for PF13354 with a fixed length of 214 positions, including 7 alignment gaps, and ensured that the wild-type sequence used in the original Ostermeier experiment was included in the alignment. Data relative to the study of the DMS of Beta-Lactamase is available at `https://github.com/francescocaredda/FeatureDCAData`

This processed MSA was used to train FeatureDCA and to compute the in-silico mutational scores. When comparing to the experimental DMS data, we excluded mutations involving missing data (i.e., those with an `inf` label) as well as trivial identity mutations (e.g., $a \to a$), which would artificially inflate correlation but carry no functional meaning.

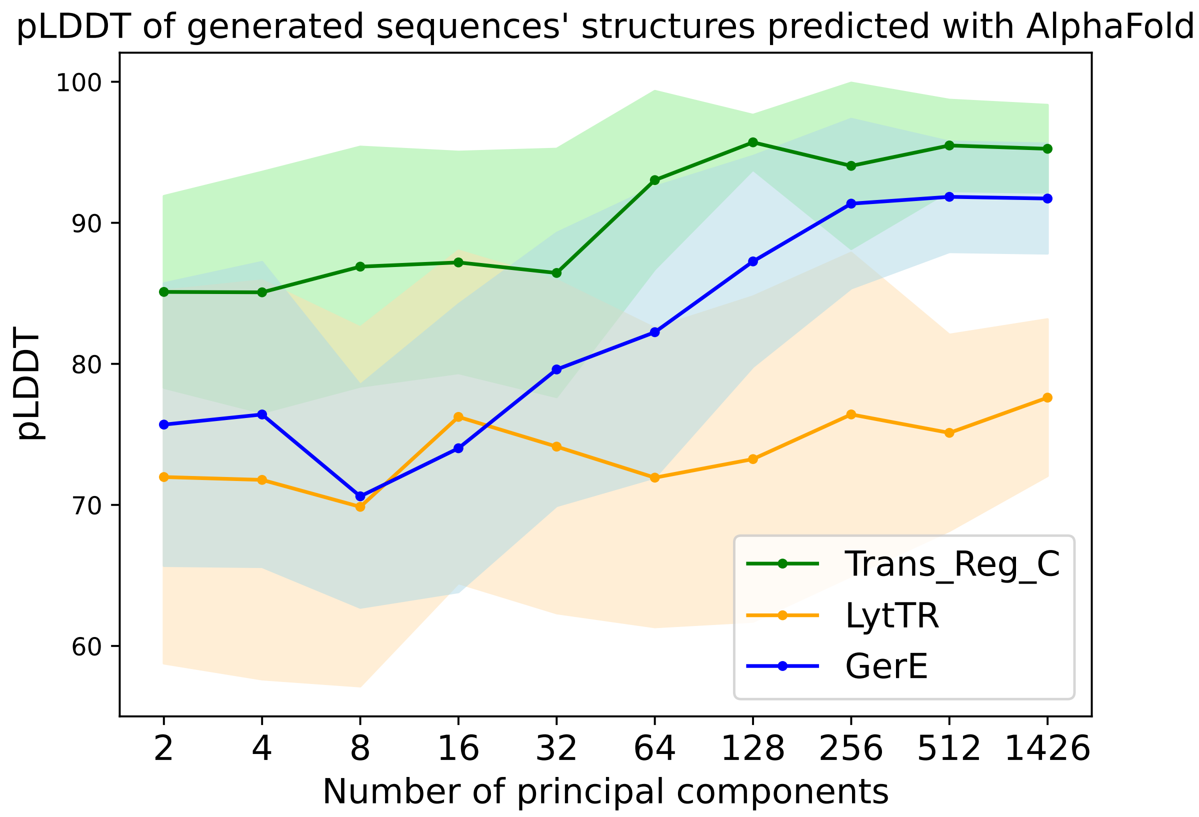

FIG. A2. **Mean pLDDT of AlphaFold-predicted structures for generated sequences.** Mean per-residue pLDDT values are shown as a function of the number of principal components used for conditioning, averaged over generated sequences for each protein family. Shaded regions indicate the standard deviation across sequences. Consistently high pLDDT values across families and conditioning dimensions indicate that the observed RMSD trends are not driven by low-confidence structure predictions.

APPENDIX A10: SUPPLEMENTARY FIGURES

The following figures complement the main text analysis on conditioned generativity. While the main text focuses on family PF13354, the figures below report analogous analyses for four additional protein families: PF00014, PF00072, PF00076, and PF00595. These include principal component (PC) projections, pairwise correlation statistics, effective sequence diversity, and structural fidelity measures for sequences generated by FeatureDCA under different conditioning schemes.

- **Figure A3**: Pearson correlation between single-site (natural vs. generated), connected pairwise correlations (natural vs. generated), and Effective depth (sequence variability) of generated MSAs as a function of the number of principal components used during training and generation. These results correspond to those presented for PF13354 in Figs 2C and 2D of the main text.
- **Figure A4**: Single-site amino acid frequency agreement between natural and generated sequences for PF13354 as a function of conditioning dimension d, shown as scatter plots with Pearson correlation coefficients.
- **Figure A5**: Wasserstein distances between the PCA distributions of natural and generated sequences for different models, using Sinkhorn divergence as described in Supplementary Section D. These results parallel the PCA-matching trends shown in Fig 2B of the main text.
- **Figure A6**: Projection of natural and generated sequences in the PCA space. This figure extends the PC projection plots shown for PF13354 in Fig 2A of the main text.
- **Figure A7**: Hamming distance and PC distance between generated and natural sequences for the four families, complementing the analysis of sequence diversity shown in Fig 3 of the main text for PF13354.
- **Figure A8**: Structural distance between generated and experimental wildtype structures for the four families, complementing the analysis of structural accuracy shown in Fig 4 of the main text for PF13354.

[1] F. Morcos, A. Pagnani, B. Lunt, A. Bertolino, D. S. Marks, C. Sander, R. Zecchina, J. N. Onuchic, T. Hwa, and M. Weigt, Proceedings of the National Academy of Sciences **108**, E1293 (2011).
[2] S. Cocco, C. Feinauer, M. Figliuzzi, R. Monasson, and M. Weigt, Reports on Progress in Physics **81**, 032601 (2018).
[3] D. C. Liu and J. Nocedal, Mathematical Programming **45**, 503 (1989).
[4] S. G. Johnson, "The {NLopt} nonlinear-optimization package," (2007).
[5] J. Feydy, T. Séjourné, F.-X. Vialard, S.-i. Amari, A. Trouvé, and G. Peyré, "Interpolating between Optimal Transport and MMD using Sinkhorn Divergences," (2018), arXiv:1810.08278 [math].
[6] J. Trinquier, G. Uguzzoni, A. Pagnani, F. Zamponi, and M. Weigt, Nature Communications **12**, 5800 (2021).
[7] S. R. Eddy, PLOS Computational Biology **7**, e1002195 (2011).
[8] A. J. Riesselman, J. B. Ingraham, and D. S. Marks, Nature Methods **15**, 816 (2018).
[9] C. E. Gonzalez, P. Roberts, and M. Ostermeier, Journal of Molecular Biology **431**, 2320 (2019).

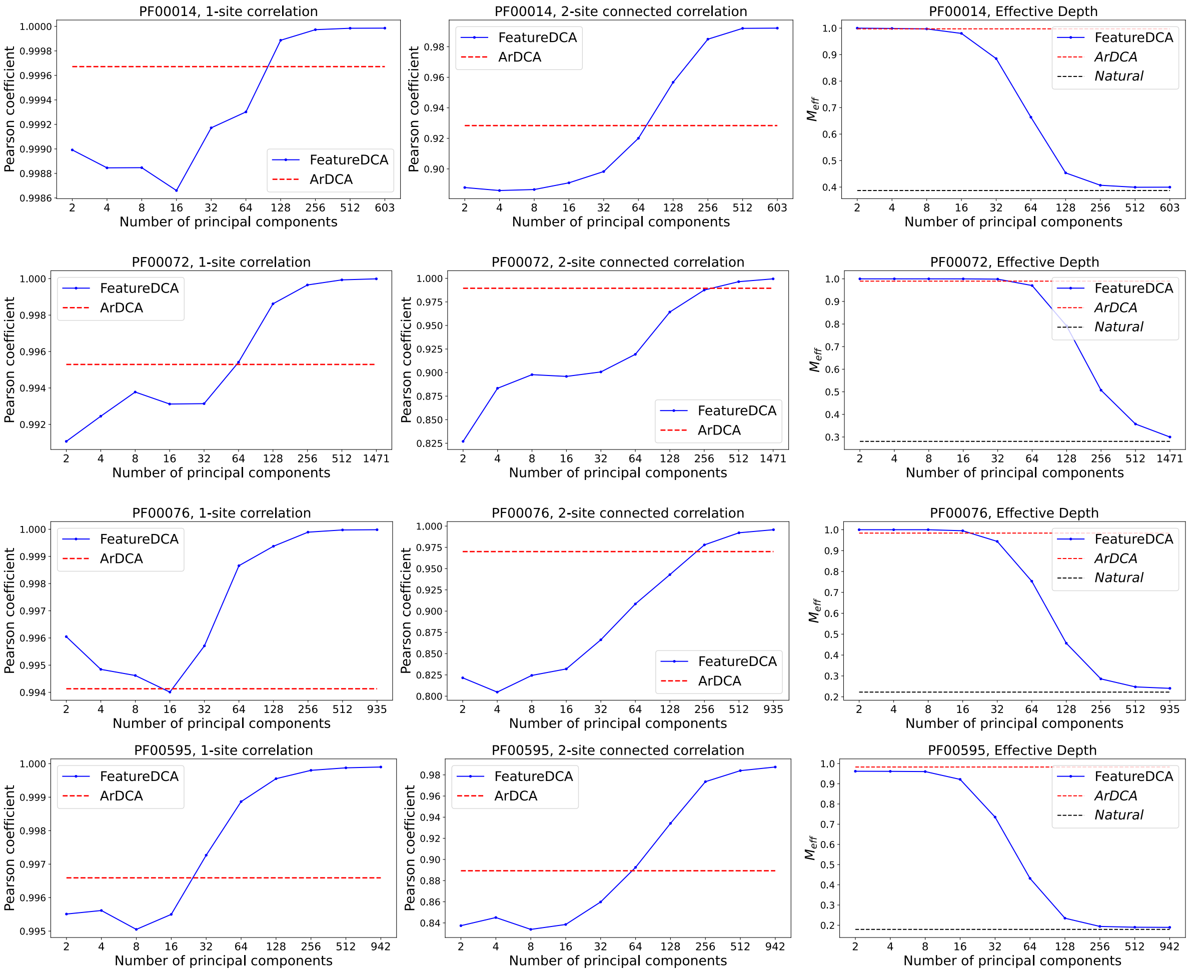

FIG. A3. **Comparison of generative and statistical properties across protein families as a function of PCA feature dimensionality**. Each row corresponds to a different protein family (PF00014, PF00072, PF00076, PF00595). **Left column**: Pearson correlation between natural and generated single-site amino acid frequency matrices, shown as a function of the number of principal components used during training and generation (blue line: FeatureDCA). **Middle column**: Pearson correlation between the pairwise connected correlations computed on natural sequences and on sequences generated by FeatureDCA, plotted as a function of the number of principal components (blue line). The red dotted line indicates the baseline correlation obtained from ArDCA. **Right column**: Effective depth (i.e., sequence diversity) of generated datasets as a function of the number of principal components (blue line: FeatureDCA). The red dotted line shows the ArDCA baseline, and the black dotted line marks the effective depth of the natural MSA.

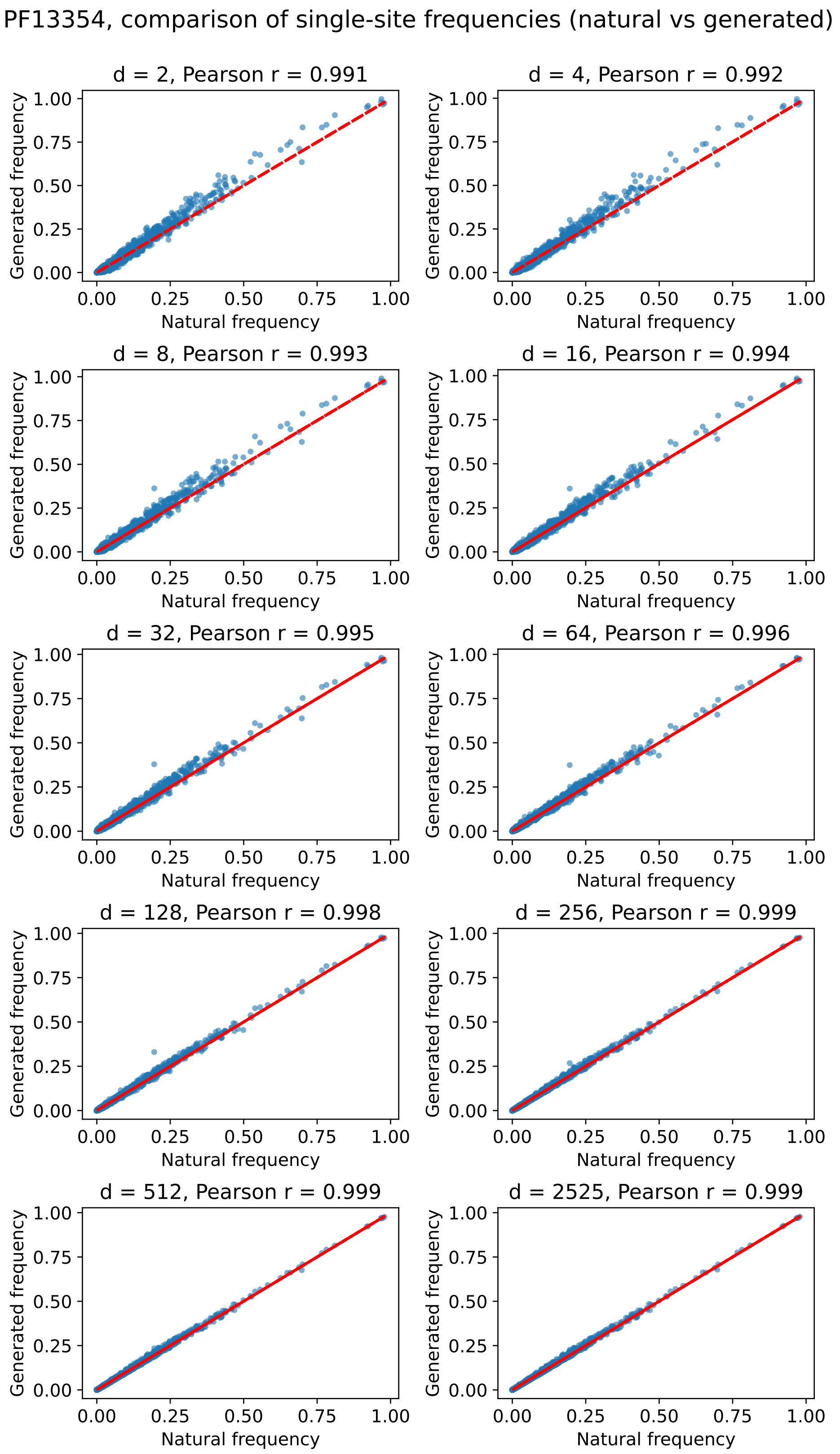

FIG. A4. **Single-site amino acid frequency comparison for PF13354.** Scatter plots comparing natural and generated single-site amino acid frequencies for the PF13354 family, shown for increasing values of the conditioning dimension d (row-wise, from top to bottom and left to right). Each point corresponds to a position–amino acid pair (i, a), with the x-axis reporting the empirical frequency $f_i^{\mathrm{nat}}(a)$ measured from the natural multiple sequence alignment and the y-axis the corresponding frequency $f_i^{\mathrm{gen}}(a)$ estimated from generated sequences. The dashed red line indicates the identity function as a reference. Titles report the Pearson correlation coefficient between natural and generated frequency matrices for each value of d. The consistently high correlations indicate that basic single-site statistics are well reproduced already at low conditioning dimension, in agreement with the higher-order correlation analyses presented in the main text.

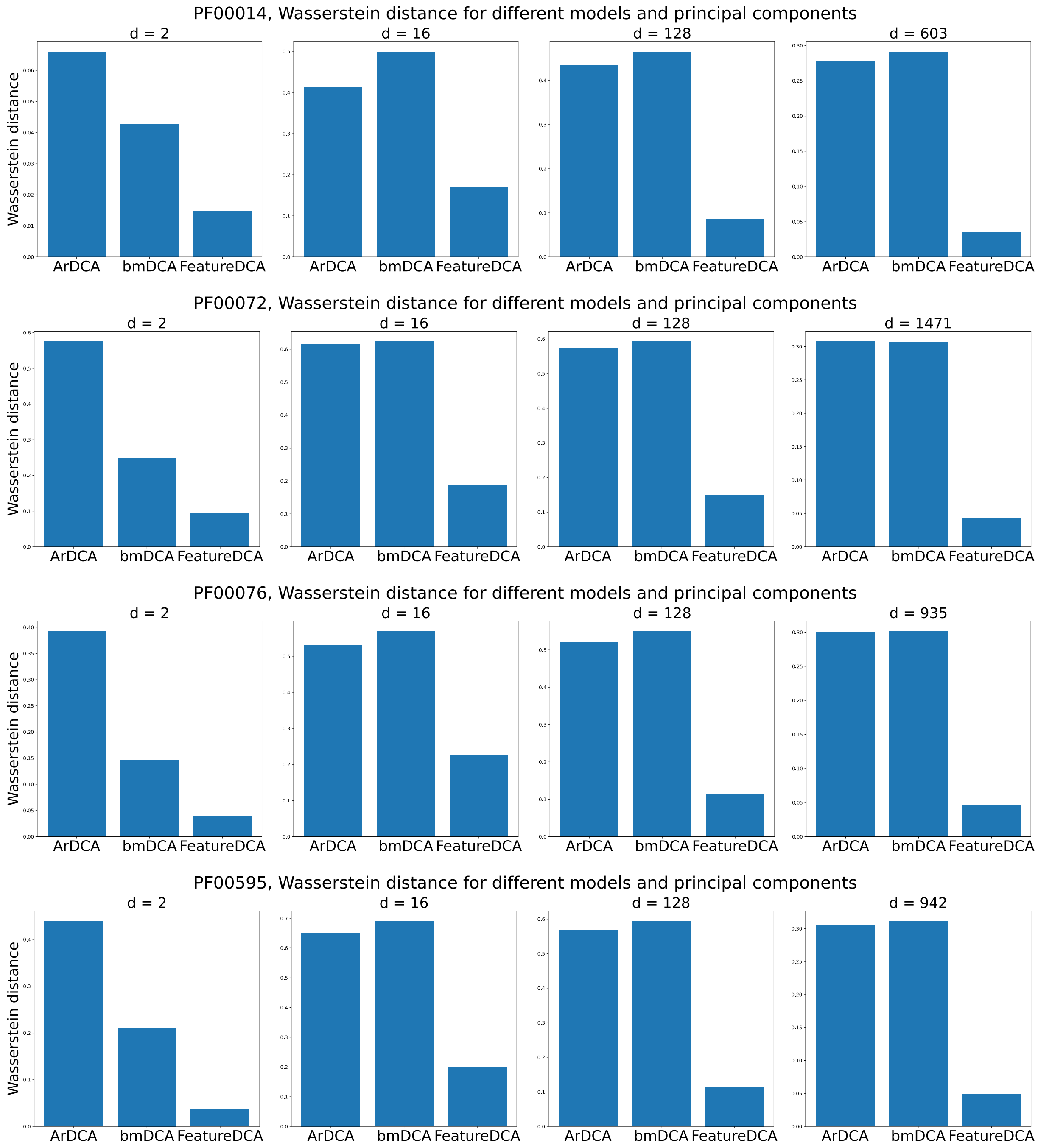

FIG. A5. **Histogram showing the Wasserstein distance between the d-dimensional PCA distributions of the natural and generated MSA for different models**. Each row corresponds to a different protein family (PF00014, PF00072, PF00076, PF00595). FeatureDCA was trained with the corresponding number of principal components used to compute the Wasserstein d-dimensional distance.

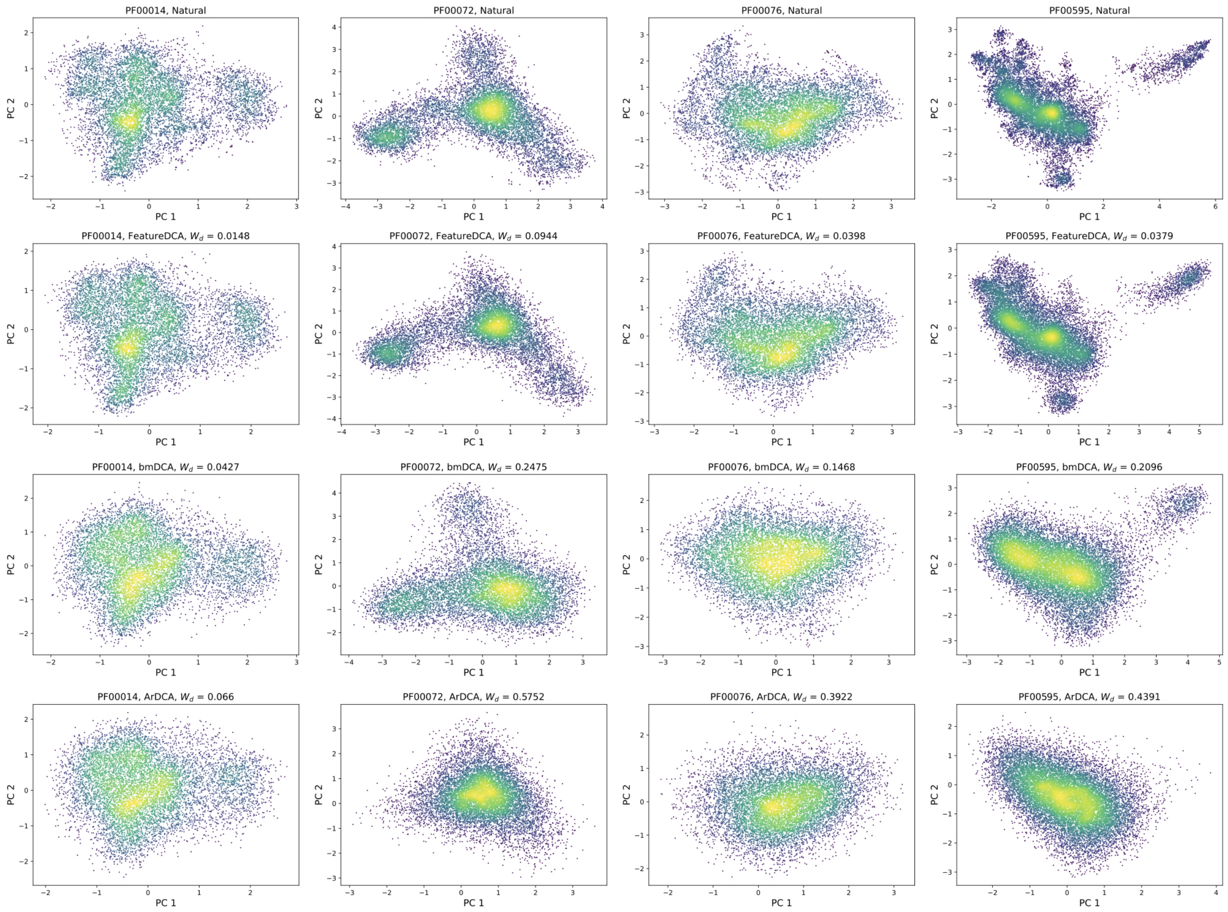

FIG. A6. **Projection onto the first two principal components of natural and generated MSAs for different protein families.** Each column corresponds to a different protein family (PF00014, PF00072, PF00076, PF00595). The first row shows the PCA projection of natural sequences, while the subsequent rows show the projections of sequences generated by FeatureDCA, bmDCA, and ArDCA, respectively. For each model and family, the Wasserstein distance W_d quantifies the discrepancy between the natural and generated distributions along the first two principal components.

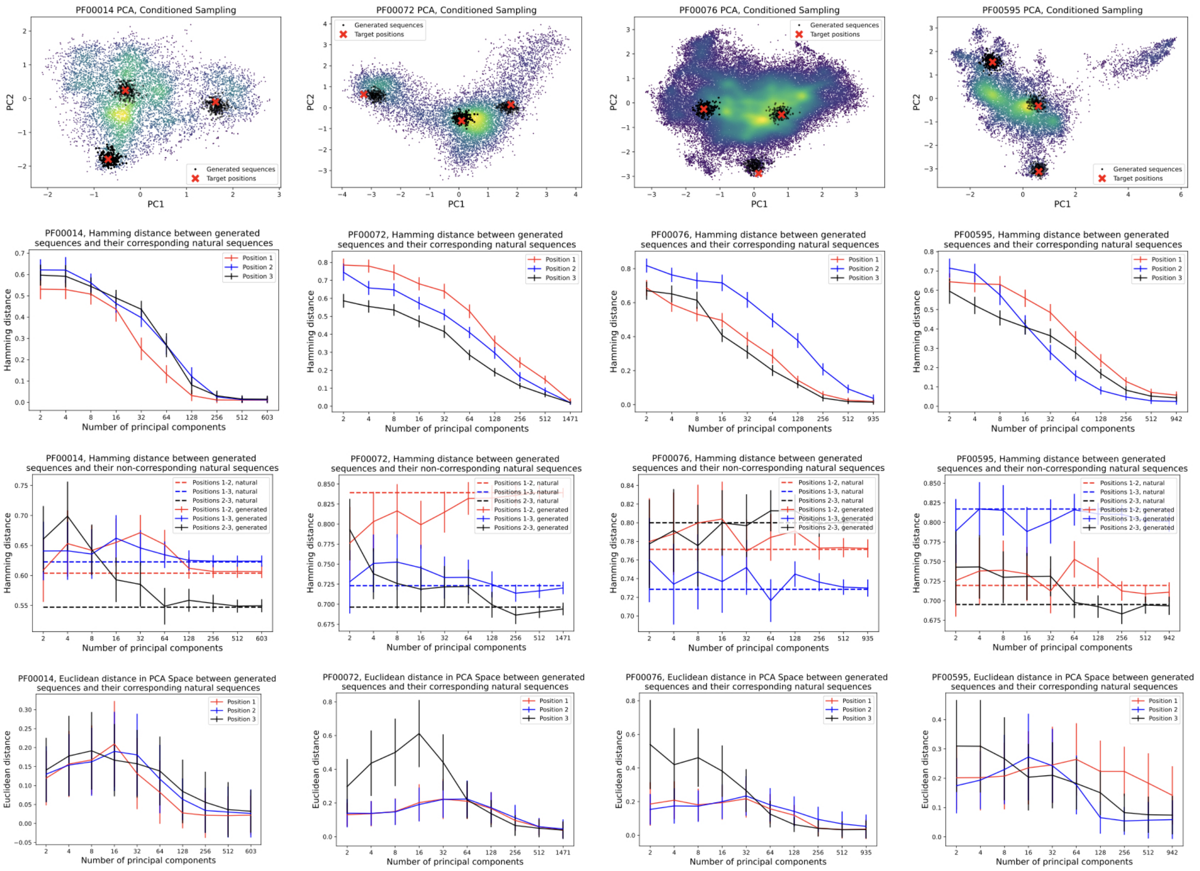

FIG. A7. **Statistical analysis of the generativity conditioned on three different positions on the PC space as a function of the number of principal components learned during training.** Each column corresponds to a different protein family (PF00014, PF00072, PF00076, PF00595). **First row**: red crosses represent the three positions chosen on different islands of the PC projection to study the different conditioned sampling. The clouds of black dots represent the generated sequences around the target positions. **Second row**: Hamming distance between the target positions and the generated sequences conditioned on those positions. **Third row**: Hamming distance between the generated sequences and the non-corresponding target positions. **Forth row**: Euclidean distance in the first two PC plane between the target positions and the generated sequences conditioned on those positions.

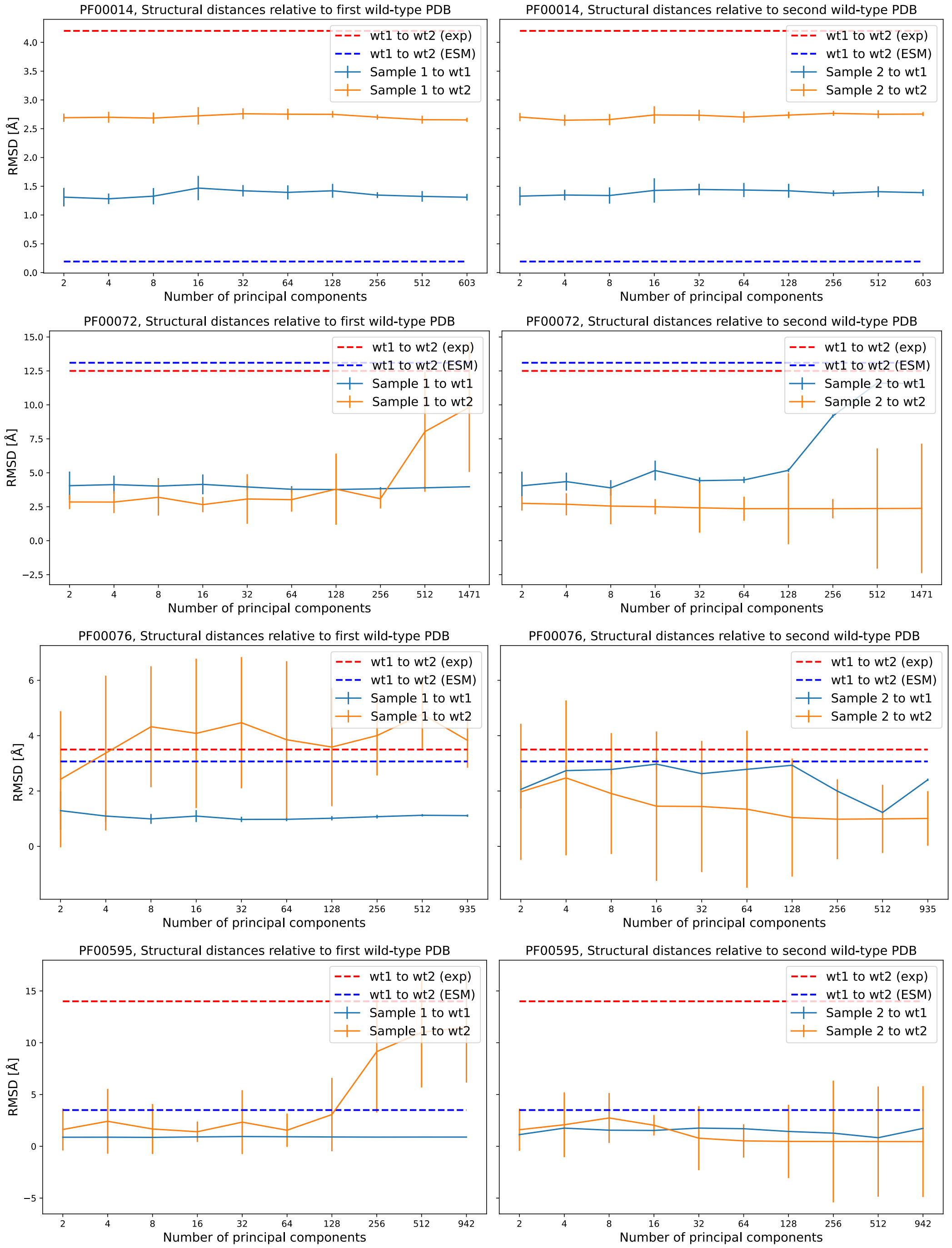

FIG. A8. **Structural similarity of generated sequences to wild-type structures for different protein families**. Each row corresponds to a different protein family (PF00014, PF00072, PF00076, PF00595). Each panel shows the RMSD (Å) between the ESMFold predicted structures of generated sequences and their respective wild-type references, plotted against the number of principal components used for learning and sequence generation. **Left**: RMSD relative to the first wild-type (wt1) structure; **Right**: RMSD relative to the second wild-type (wt2) structure. Dashed lines indicate the RMSD between the two wild types using experimental structures (red) and ESMFold predictions (blue). Solid lines represent average RMSDs of generated samples to wt1 (blue) and to wt2 (orange), with error bars showing standard deviation.
