# Supplementary material for: Controllable protein design via autoregressive direct coupling analysis conditioned on principal components
Source: PLoS Comput Biol. 2026 Feb 19;22(2):e1013996. doi: 10.1371/journal.pcbi.1013996 (PMC12935303; doi:10.1371/journal.pcbi.1013996)
Supplement: Attachment — Submitted filename: Risposte Reviewers PLoS.pdf [file pcbi.1013996.s002.pdf]

**RESPONSE TO REVIEWERS**

Please find enclosed the revised manuscript by Francesco Caredda, Lisa Gennai, Paolo De Los Rios, and Andrea Pagnani titled *"Controllable protein design via autoregressive Direct Coupling Analysis conditioned on principal components".* We have carefully considered the Reviewers' comments and amended the text accordingly.

In response to both Reviewer 1 and Reviewer 2, we have included several clarifications and additional analyses to improve the clarity, contextualization, and robustness of the manuscript. These updates are primarily in the Introduction and Methods sections, and in the Supporting Information. In particular, we moved the details of the DMS analysis from the SI to the main text.

Below, you will find our detailed responses (in blue) to each of the reviewers' comments.

---

---

We sincerely thank Reviewer 1 for a careful and thorough review of the manuscript. The comments and insightful questions provided, including drawing our attention to the classical results of Sanders and Schneider and encouraging a deeper analysis of the RMSD discrepancies observed for PF00014, have been instrumental in improving the clarity and depth of the manuscript and in strengthening the organization of the Supplementary Materials.

**REVIEWER 1:**

The manuscript describes an extension of the standard DCA, routinely used in connection with structure prediction of proteins, to include annotated functional data. In my opinion, this is a very relevant study that further improves a technique which has already proven seminal in the field of computational biology. Therefore, I recommend its publication with minor revision, mainly focused on the clarity of the text.

1. "Lack of control over the output distribution" (line 26) seems really pessimistic; maybe it is not exactly what the authors meant to say.

We agree that the original phrasing could be interpreted as overly pessimistic. Our intention was not to suggest a fundamental inadequacy of generative DCA models, but rather to highlight a practical limitation of standard unconditional sampling, which does not directly allow steering generation toward specific, user-defined properties without additional conditioning mechanisms. We have revised the text to clarify this point and to emphasize that the limitation concerns the default sampling regime rather than the modeling framework itself.

2. Little is said in the main text of the functional features that are included in the model (the y_k quantities). It could be beneficial to the reader to have a clear idea of what they are from the very beginning.

The original idea was to highlight the flexibility of the model in the choice of the feature vectors. However, we agree that the definition of the conditioning features y_k was introduced too late in the original version of the manuscript. To improve clarity and accessibility, we have revised the main text to explicitly describe the nature of the y_k quantities at their first introduction. In particular, in the paragraph following Eq. 2 in the Method Section, we now clarify that, in the present work, the feature vector y_k corresponds to the principal component projections of sequences in the multiple sequence alignment, and we provide intuition about their role as low-dimensional descriptors of dominant sequence variability. This change should ensure that readers have a clear understanding of the conditioning features from the beginning, with a mention of the possible generalizations at the end of the Methods section.

3. As usual, the gaps are treated as a type of amino acid, but I would find it strange they contribute actively to defining function, as eq. 2 would suggest. Do they obey some special rule?

We thank the reviewer for raising this point. Although gaps do not directly encode protein function in the same way as amino acids, they carry alignment- and coevolution-related information associated with insertion/deletion (indel) patterns, which can reflect structural or functional constraints. For this reason, including gaps is important for a consistent statistical description of the protein family and for capturing correlations between sequences and conditioning features.

In the Potts/DCA framework, gaps do not obey any special rule: during parameter learning they are treated as an additional symbol, exactly as in standard DCA formulations. Finally, it is worth noting that in standard DCA, gaps are typically included during parameter inference but are often excluded when computing downstream quantities such as contact scores. In our generative setting, however, explicitly including gaps is essential to sample correctly and meaningfully from the learned sequence distribution.

4. A classic work by Sanders and Schneider (Proteins Structure. Funct. Gen. 9, 56, 1991) defines a curve in the space of hamming distance/sequence length that separates homologs from pairs with unknown relation, saturating at distance 0.75 for long chains. Can the maximum Hamming distance found in the generated sequence (line 179) related to that?

We are very grateful to the reviewer for pointing out this classical and highly relevant result, which we were not aware of at the time of writing. Indeed, the saturation of the Hamming distance at approximately 75% for long protein chains provides a natural statistical reference for interpreting the maximum distances observed in our generated sequences, particularly in the regime of small conditioning (for $d = 2, 4$). We have revised the manuscript to explicitly mention this result and to note that our observations are consistent with the findings of Sanders and Schneider.

5. Predictions are compared with experimental structure with a RMSD (line 200), but it is well known that RMSD displays very nonlinear effects, structural variations producing large changes in the RMSD when this is small and negligible changes when it is large. Can the mismatch observed in the case of PF00014 be analysed in terms of number of common contacts, defined in some appropriate way?

We agree that RMSD alone may be misleading as a measure of structural similarity, and we therefore performed additional analyses for the PF00014 family using topology-aware metrics. In particular, we complemented the RMSD comparison with both the TM-score and an analysis of structural contact overlap, where contacts are defined as pairs of residues more than 4 positions apart,  with their alpha-carbons at distances smaller than 8 Å.

The experimental structures considered for this analysis (PDB IDs 1UUB and 1LD6) are both well-established structures for the PF00014 family, determined under standard experimental conditions. In particular, 1LD6 corresponds to a heavily mutated variant of the wild-type bovine pancreatic trypsin inhibitor (4PTI), obtained by introducing eight alanine substitutions, while 1LD5 represents a closely related variant carrying a single mutation. Focusing on the 1LD6 case, we find that the AF/ESM-predicted structure of the 1LD6 sequence is significantly closer to the wild-type and minimally mutated structures of the family than to the experimentally determined 1LD6 structure itself. Specifically, the TM-score between the predicted structure and the experimental 1LD6 structure is approximately 0.69, whereas higher TM-scores are obtained when comparing the same predicted structure to 1LD5 and to the wild-type 4PTI (0.85 and 0.98, respectively). Consistently, the number of shared contacts between the predicted structure and 1LD6 is lower than the number of shared contacts with 1LD5 and 4PTI (91 against 110 and 119, respectively).

These results indicate that the observed discrepancy is not an artifact of the nonlinear behavior of RMSD, but rather reflects a genuine limitation of AF/ESM in reproducing the experimentally observed fold of this heavily mutated construct. We have added a short discussion of these additional analyses to the revised manuscript and provided full details in the Supporting Information.

6. I would have preferred to see some results concerning mutations (line 288) in the main text. Is it possible to distinguish between effects of mutations on thermodynamic stability and on function (I.e., all the rest)?

In response, we have moved the figure relative to the DMS results from the Supporting Information to the main text. Concerning the distinction between thermodynamic stability and functional effects, DCA-based models, including FeatureDCA, do not explicitly disentangle these contributions. Instead, they assign mutations a likelihood-based score that reflects a statistical fitness signal, consistent with how experimental DMS measurements are typically interpreted. We have clarified this point explicitly in the revised manuscript.

---

---

We sincerely thank Reviewer 2 for their careful reading of the manuscript and for their valuable suggestions. Their insightful comments have helped us refine the presentation, clarify the model's aims, and strengthen the discussion of the analyses and results.

**REVIEWER 2:**

Minor Comments

Overall, the manuscript presents an interesting approach for conditioning autoregressive protein sequence models on PCA-derived features. The method is clearly explained at a high level, and the comparisons against existing DCA-based models are valuable. I have several minor comments that, if addressed, would improve clarity, reproducibility, and contextual grounding of the work.

1. Clarification of model architecture

The manuscript refers to the use of an autoregressive model but does not describe the underlying architecture. Please specify whether the model is transformer-based, attention-based, convolutional, or uses another design, and include the total number of parameters or layers.

In our manuscript, the term *autoregressive* refers to the probabilistic factorization of the joint sequence distribution along the protein chain (Eq. 1 in the main text), rather than to a specific deep neural network architecture (e.g., Transformers, attention-based, or convolutional models). In our approach, each conditional distribution is parameterized explicitly by a shallow energy-based Potts model with pairwise couplings in sequence space and interactions with latent variables, as in Eq. 2, following the ArDCA framework. Model parameters are learned in a family-dependent manner by maximum-likelihood estimation. We have revised the Methods section to clarify this distinction and have added a statement describing how the number of model parameters scales with sequence length.

2. Dataset sizes and splitting strategy

The authors should provide explicit details on the size of the training MSAs (number of sequences, average length), the size of the evaluation datasets, and the procedure used to split or select data for training and evaluation. Clarifying whether they rely on standard Pfam splits or their own selection criteria would improve experimental transparency.

We thank the reviewer for this comment. To improve transparency, we have added a summary table in the Supporting Information Appendix S4 that reports, for each protein family studied, the number of sequences, sequence length, and number of parameters used to train the model. Our model is trained in an unsupervised manner by maximizing the likelihood over the full multiple sequence alignment using a quasi-Newton optimization algorithm (L-BFGS), which is run until convergence. As a result, no explicit training/validation/test split is required.

For consistency, we relied on MSAs previously curated in the ArDCA study when available, and we performed our own curation for the analyses on PF00072 and for the deep mutational scanning benchmark on PF13354. Full details of dataset construction and

preprocessing were already provided and are now clearly referenced in the Supporting Information.

3. Discussion of recent MSA-based generative models
The introduction would benefit from situating the method within the context of more recent MSA-based generative models such as PoET-2 and Tranception, in addition to bmDCA and ArDCA. A brief comparison or rationale for focusing on DCA-based baselines would help readers understand the positioning of this work.

We agree that placing our approach in the context of more recent MSA-based generative models is useful, and we have revised the Introduction accordingly to explicitly mention Tranception and PoET-2 and to further clarify the aim of our paper. In particular, these methods rely on deep transformer-based architectures with hundreds of millions of parameters and have been primarily developed to achieve strong performance in mutational effect prediction and sequence scoring. In contrast, our interest lies in shallow, interpretable, generative statistical models. In particular, we deliberately focus on DCA-based approaches to extend a well-established and seminal framework, with the goal of enabling explicit and controllable sequence generation while preserving analytical tractability and interpretability. For these reasons, bmDCA and ArDCA constitute the most appropriate reference points for positioning and evaluating the proposed method.

4. Structural prediction confidence metrics
The structural evaluation relies on RMSD comparisons between predicted and experimental structures. It would be helpful to also report pLDDT or other confidence metrics from AlphaFold 3 or ESMFold when folding generated sequences.

We thank the reviewer for this suggestion. We agree that confidence metrics provide valuable complementary information to RMSD-based structural comparisons. We have therefore analyzed the predicted Local Distance Difference Test (pLDDT) scores associated with AlphaFold predictions of generated sequences for the RR homodimer families considered in the structural evaluation. This analysis is now reported in SI Appendix S8 and referenced in the main text. It shows that predicted structures are generally associated with moderate to high pLDDT values across conditioning dimensions. Moreover, differences observed between RR homodimer classes in terms of pLDDT are consistent with the trends reported in the RMSD-based analysis in Figure 7.

5. Amino acid composition analysis
To further validate the generative capabilities of the model, the authors could provide amino acid frequency distributions for natural versus generated sequences. This would allow readers to assess whether the model captures basic sequence-level statistics in addition to PCA and Hamming distance metrics.

We note that the manuscript already reports a quantitative comparison of amino acid frequency distributions. In particular, Figure 2 in the main text and Figure S2 (now Figure S3) in the Supporting Information show the Pearson correlation coefficient of pairwise connected correlations between natural and generated sequences. Connected correlations usually provide a stronger and more informative metric than single-site amino acid frequencies

alone, as agreement at the level of connected correlations usually implies that single-site statistics are also well captured, up to finite-sample fluctuations.

Nevertheless, to provide a direct and transparent assessment of basic sequence-level properties, we have extended the Supporting Information to include an explicit analysis of single-site amino acid frequencies. We emphasize that such an analysis is well defined in the case of global generation, where the natural reference distribution is given by the empirical MSA, but is not meaningful for “in-place generation”, where sampling is conditioned on a specific point in feature space and the natural reference often corresponds to a single sequence. For this reason, we present the single-site frequency analysis as a complementary supporting analysis associated with the global-generation results shown in Figure 2.

Specifically, we report the Pearson correlation between natural and generated single-site frequency matrices as a function of the conditioning dimension “d” for all protein families considered, as shown in Figure S3. In addition, we include a detailed scatter-plot comparison for the PF13354 family as a representative example. For reasons of space and clarity, we chose to summarize the single-site agreement using correlation coefficients and to show a detailed scatter plot for a single family only. Extending such visualizations to all families and all values of “d” would have resulted in a large number of redundant panels and substantial clutter, without providing additional insight beyond the quantitative trends already reported.

These results confirm that amino acid composition is well reproduced across conditioning regimes, in full agreement with the higher-order correlation analyses presented in the main text.

6. Details of DMS evaluation
For the DMS experiments, please report the number of mutations or samples included and provide the performance metrics of each model (FeatureDCA, bmDCA, ArDCA) directly in the main text. This would help clarify the extent and rigor of the DMS evaluation.

In response to this, in the revised manuscript, we have expanded the main text to explicitly describe the deep mutational scanning benchmark, which comprises all single-amino-acid substitutions with available experimental fitness measurements for the TEM-1 beta-lactamase protein. Quantitative performance metrics, reported as Spearman rank correlations between predicted and experimental mutational effects, are now provided directly in the main text for both FeatureDCA and ArDCA, and the corresponding figure has been moved from the Supporting Information to the main text. Additional details on dataset curation and preprocessing are provided in the Supporting Information

We did not include a bmDCA benchmark in this study. However, ArDCA, on which FeatureDCA is directly based, has been previously benchmarked against plmDCA and other generative models in the original ArDCA study (Trinquier et al.). In this context, ArDCA serves as a natural baseline for assessing the impact of the conditioning extension proposed in our work.

Once again, we sincerely thank the Reviewers for their time and insightful comments. We remain open to any further suggestions or feedback as needed.

Sincerely,

Francesco Caredda
Lisa Gennai
Paolo De Los Rios
Andrea Pagnani
